# Bottlebrush Polymers for Articular Joint Lubrication: Influence of Anchoring Group Chemistry on Lubrication Properties

**DOI:** 10.1021/acsami.4c07282

**Published:** 2024-07-09

**Authors:** Karolina Turczyńska, Mahdi Rahimi, Gholamreza Charmi, Duy Anh Pham, Hironobu Murata, Marcin Kozanecki, Paulina Filipczak, Jacek Ulański, Tadeusz Diem, Krzysztof Matyjaszewski, Xavier Banquy, Joanna Pietrasik

**Affiliations:** † Department of Molecular Physics, Faculty of Chemistry, Lodz University of Technology, Zeromskiego 116, 90-924 Lodz, Poland; ‡ Orthopedic Research Laboratory, Hôpital du Sacré-Coeur de Montréal, Université de Montréal, H4J 1C5 Montréal, QC, Canada; § Institute of Polymer and Dye Technology, Faculty of Chemistry, Lodz University of Technology, Stefanowskiego 16, 90-537 Lodz, Poland; ∥ Canada Research Chair in Bio-inspired Materials and Interfaces, Faculty of Pharmacy, Université de Montréal, C.P. 6128, succursale Centre Ville, Montréal Qc H3T1J4, QC, Canada; ⊥ Department of Chemistry, Carnegie Mellon University, 4400 Fifth Avenue, 15213 Pittsburgh, Pennsylvania, United States; # Collegium Civitas, Plac Defilad 1, 00-901 Warsaw, Poland

**Keywords:** bottlebrushes, articular cartilage, biolubrication, friction coefficient, osteoarthritis

## Abstract

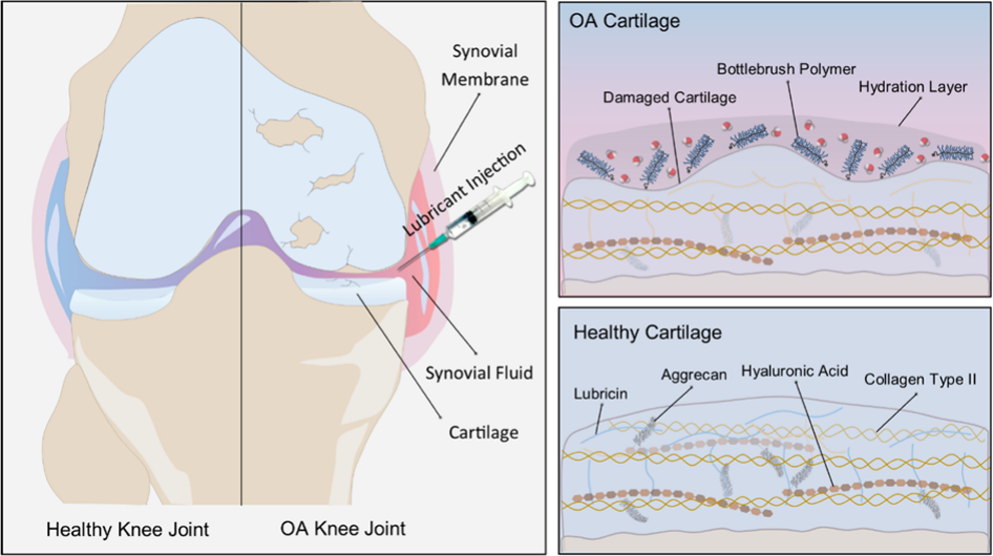

The role of carboxylic, aldehyde, or epoxide groups incorporated into bottlebrush macromolecules as anchoring blocks (or cartilage-binding blocks) is investigated by measuring their lubricating properties and cartilage-binding effectiveness. Mica modified with amine groups is used to mimic the cartilage surface, while bottlebrush polymers functionalized with carboxylic, aldehyde, or epoxide groups played the role of the lubricant interacting with the cartilage surface. We demonstrate that bottlebrushes with anchoring blocks effectively reduce the friction coefficient on modified surfaces by 75–95% compared to unmodified mica. The most efficient polymer appears to be the one with epoxide groups, which can react spontaneously with amines at room temperature. In this case, the value of the friction coefficient is the lowest and equals 0.009 ± 0.001, representing a 95% reduction compared to measurements on nonmodified mica. These results show that the presence of the functional groups within the anchoring blocks has a significant influence on interactions between the bottlebrush polymer and cartilage surface. All synthesized bottlebrush polymers are also used in the preliminary lubrication tests carried out on animal cartilage surfaces. The developed materials are very promising for future *in vivo* studies to be used in osteoarthritis treatment.

## 1 Introduction

Osteoarthritis (OA) is the most common type of arthritis and one of the most common causes of disability. It is a degenerative joint disease that affects multiple components, including synovial fluid and cartilage. Due to the complexity of genetic, metabolic, and environmental factors, the mechanism of degradation has remained a subject of intense research.^1−3^ The disease can affect many joints, including the knee, hip, hand, cervical, or temporomandibular.^4,5^ Its most common symptoms include joint dysfunction, pain, and stiffness.^6^ Several symptom management approaches have been developed in OA treatment. When simple lifestyle and self-management methods such as physical therapy or weight loss fail, pharmaceutical managements are introduced. In this approach, medications like acetaminophen, nonsteroidal anti-inflammatory drugs, opioids, and corticosteroid injections are often used to reduce pain and inflammation in joints, among others.^7,8^ When pharmaceutical methods are not effective, the patient is qualified for surgical treatment such as joint repair or joint replacement.

The complex structure of the joint allows movement and reduction of the effects associated with friction during movement.^9,10^ Muscles and ligaments are responsible for stabilization, whereas the cartilage tissue and synovial membrane provide the appropriate friction environment. A large extracellular matrix surrounding the chondrocytes, the only cells found in healthy cartilage, gives cartilage its properties, such as compression resistance and the ability to absorb shocks under loads.^11^ The synovial membrane maintains a fluid-filled space and provides a lubricating environment.^12^ The synovial membrane is also responsible for the production of synovial fluid, which affects the reduction of the coefficient of friction between cartilage tissues. A change in synovial fluid content negatively affects the metabolism of articular cartilage and causes greater cartilage wear.^13,14^

One aspect of osteoarthritis is the progressive reduction of cartilage tissue, which causes significant difficulty in the proper movement of joint surfaces. The ability to regenerate cartilage is limited. The newly formed tissue has worse mechanical properties due to its fibrous structure.^15^ Deterioration of mechanical properties of newly formed fibrous cartilage in osteoarthritis implies a combination of reduced elasticity and compliance, increased surface roughness, decreased durability, and altered biomechanical function. These changes lead to impaired shock absorption, increased friction and wear, greater susceptibility to damage, and overall decreased effectiveness in maintaining joint health and function.

Cartilage contains proteoglycans and hyaluronic acid produced by type B synoviocytes and chondrocytes. As the pressure between two cartilage surfaces increases and fluids could be squeezed out, there is a need for a special lubrication mechanism. The boundary cartilage lubrication involves the formation of an adhesive layer that allows the maintenance of the distance between friction surfaces and reduces the adhesion.^16,17^ This layer mainly contains aggrecans, phospholipids, hyaluronic acid, and lubricin. Lubricin is produced by chondrocytes and synoviocytes and plays an essential role in the lubrication of articular cartilage. It is responsible for controlling inflammation, stopping cartilage wear and tear as well as adhesion and proliferation of synovial cells.^18^ Lubricin also has the potential to be used as an agent that prevents bacterial proliferation by reducing adhesion and growth on the surface of model tissues.^19^ The lack of lubricin between the cartilage surfaces induces the adhesion growth that causes the stick–slip phenomenon.

**Figure 1:**
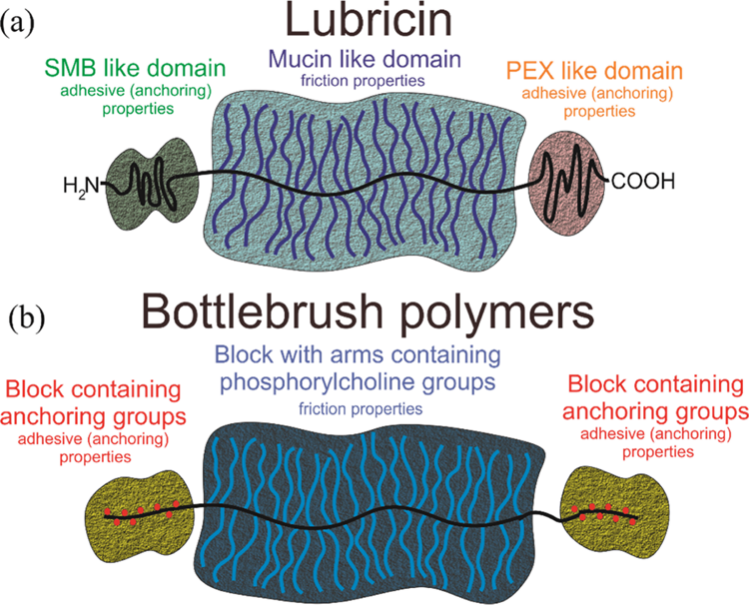
Scheme of natural lubricin (a) and bottlebrush polymer (b).

Lubricin, a heavily O-glycosylated protein, has a triblock bottlebrush structure consisting of a mucin-like central domain and two nonglycosylated ends (Figure 1a). The negatively charged central domain is hydrophilic and forms a hydration layer that provides lubricating and antiadhesion properties.^20^ The C- or N-end domains physicochemically interact with the cartilage surface. Grafted and fully extended structures create repelling lubricating layers. The steric repulsion forces between two layers of lubricin on the opposing cartilages also involve hydration forces.

Effective pharmaceuticals dedicated to early OA treatment have not yet been available on the market. However, some formulations were proposed for the reduction of pain or inflammation. Treatment of symptoms does not eliminate the cause of the disease progression and that is why it is crucial to formulate artificial joint lubricants. These are injectable viscous substances based on hyaluronic acid (HA) such as cross-linked hyaluronic acid, sodium hyaluronate, or hyaluronan.^21^ Their main drawbacks are relatively fast enzymatic degradation, high cost due to the necessity of repeating treatment, swelling after the procedure, limited clinical efficacy, and the fact that the effectiveness of HA-based solutions often depends on the age of the patient.^22,23^ Very recently, new copolymers with molecular bottlebrush topology have been considered as lubricants for cartilage and as an interesting alternative to current formulations.^12,24−28^ Densely grafted side chains in bottlebrush domains mimic the lubricin central domains, which exhibit unique lubrication properties (Figure 1b). They affect lubrication under boundary-mode conditions that can minimize cartilage wear.

Bottlebrush copolymers can be precisely synthesized using the reversible-deactivation radical polymerization (RDRP) methods such as reversible addition–fragmentation chain transfer polymerization (RAFT)^29^ or atom transfer radical polymerization (ATRP).^30^ These methods enable the preparation of materials with desired architecture, composition, and functionality.^31^ Three strategies can be used for the synthesis of bottlebrush polymers: “grafting-to” (attachment of presynthesized side chains to the backbone), “grafting-from” (synthesis of side chains from a backbone by polymerization of monomers), and “grafting-through” (polymerization of macromonomers).^32^ Such macromolecules were already tested as potential joint lubricants.^33,34^ It is possible to design and control the molecular parameters of bottlebrushes, including molecular weight, the length of the side chains, and their density along the backbone, which enables to mimic lubricin.^33,35,36^ The presence of additional blocks in the bottlebrush structure can also control its properties. It was demonstrated before that adhesive blocks (anchoring blocks) such as quaternized poly(2-dimethylaminoethyl methacrylate-*co*-methyl methacrylate) have an impact on lubrication properties, increasing the critical pressure of the polymer film deposited on the mica surface that can reduce wear of the mica sample.^37^

The introduction of specific functional groups into the bottlebrush structure can enable stronger interactions with the cartilage surface at different stages of OA. In this work, we present the lubrication properties of new bottlebrush copolymers containing three different anchoring blocks with aldehyde, carboxylic, or epoxide groups. These functional groups were expected to react effectively with the amine groups present on the cartilage surface and therefore have an impact on the lubricating properties of the bottlebrush polymers inside the joint. The lubrication properties were evaluated using the surface force apparatus (SFA) on negatively charged mica, mica surfaces modified with amine groups, and animal articular cartilage tissue.

## 2 Experimental Section

### 2.1 Materials

Initiators and catalytic systems for RAFT and ATRP polymerization, including 2-cyanopropan-2-yl benzodithioate (CPBDT, 97% HPLC), 2,2′-azobis(2-methylpropionitrile) (AIBN, 98%), α-bromoisobutyryl bromide (BIBB, 98%), copper(I) chloride (CuCl, 99.995% trace metals basis), copper(II) chloride (CuCl_2_, 99.999% trace metals basis), and 2,2′-bipyridyl (bpy, 98%), were purchased from Merck and used as received. All monomers including 2-hydroxyethyl methacrylate (HEMA, 98%), methyl methacrylate (MMA, 98%), glycidyl methacrylate (GMA, 97%), and *tert*-butyl methacrylate (tBMA, 98%) were obtained from Merck and were purified from inhibitors by distillation under reduced pressure. 2-Methacryloyloxyethyl phosphorylcholine (MPC, 97%) was also received from Merck and used without further purification. Other reagents, including methacryloyl chloride (97%), triethylamine (TEA, 99.5%), and trifluoroacetic acid (TFA, 99%), were purchased from Merck and used as received. Two monomers including 2-(2-bromoisobutyryloxy)ethyl methacrylate (BIBEMA) and 4-formylphenyl methacrylate (FPMA) were synthesized according to recent literature and are reported in the Supporting Information (SI).^27,38,39^ All solvents such as dichloromethane (DCM), methanol, diethyl ether, *n*-hexane, and *N*,*N*-dimethylformamide (DMF) were purchased from Avantor Performance Materials Company. The material used for the lubrication experiments was a Ruby Mica V-1 with optical grade 1, purchased from S&J Trading Inc. The surfaces were modified using (3-aminopropyl)triethoxysilane purchased from Merck. The chicken knee joint cartilage tissue was selected for some tribological experiments because of its small size and common availability. Food-grade chicken legs were used.

### 2.2 Instrumentation

Fourier transform infrared spectroscopy (FTIR) was used for the characterization of the chemical structure and functional groups present in the samples. Measurements were carried out in the mid-infrared region of 500–4000 cm^–1^ with 32 scans using an FTIR Nicolet 6700 FTIR spectrometer (Thermo Fisher Scientific). The ATR accessory equipped with a single reflection diamond crystal was used for all analyses.

Nuclear magnetic resonance spectroscopy (^1^H NMR) was used to determine the chemical structure of the synthesized compounds as well as the monomer conversions during the polymerization reactions. The ^1^H NMR spectra were recorded with Bruker Advance II Plus 700 MHz and Bruker Avance DPX 250 MHz instruments using chloroform-d_3_ (CDCl_3_), or methanol-d_4_ (CD_3_OD), as the solvent.

Size exclusion chromatography (SEC) measurements were performed with a Wyatt instrument (Wyatt Technology) equipped with two perfect separation solution (PSS) columns and one guard column (GRAM Linear (10 μm, *M*_n_ between 800 and 1,000,000 Da)), a differential refractometer (RI), and light scattering (LS) detectors. Measurements were carried out in DMF as an eluent, containing 50 mmol LiBr, at a flow rate of 1 mL/min. Poly(methyl methacrylate) (PMMA) standards (*M*_n_ = between 602 and 2,200,000 Da) were used for the determination of the calibration curve. Alternatively, SEC multiangle light detector (SEC-MALS) measurements were performed using an Agilent SEC system equipped with a Waters Ultrahydrogel Linear column and coupled with MALS, UV, and RI detectors (Wyatt Technology), using PBS buffer (pH 7.4) and 100 mM sodium phosphate (pH 2.5) with 0.2 vol % trifluoroacetic acid as an eluent. The sample concentration was approximately 2 mg/mL, and the injection load was 100 μL. The refractive index increment d*n*/d*c* was determined by manual injection of the samples with varying concentrations in PBS into the RI detector.

The average hydrodynamic diameter of the synthesized polymers was measured by dynamic light scattering (DLS) using a Zetasizer NanoZS90 instrument at 25 °C. All polymers were dissolved and diluted with water to a concentration of 5 mg/mL prior to characterization.

A polymer solution with a low concentration of 25 μg/mL in Mill-Q water was prepared for deposition on mica. After allowing the polymer to adsorb in air for 1 h, the excess solution was gently removed, and the surface was carefully rinsed with water. AFM imaging was performed on a Bruker FastScan microscope using the PeakForce Quantitative Nanomechanics imaging mode. Scanasyst air tips were employed, oscillating at their characteristic frequency of around 70 kHz. The cantilever used was 115 μm long and 35 μm wide, with a typical tip radius of 12 nm and a force constant of 0.4 N/m. The scanning rate during the process was 128 scans per minute. Height channels were acquired at different Z-force levels, and the width and contour length of the polymers were analyzed using ImageJ (version 1.49).

A Surface Force Apparatus model 2000 (SFA 2000, SurForce, LLC) with a spectrometer and a digital camera (Andor Technology) was used for lubrication tests. The bimorph slider was driven by a function waveform generator Agilent 33250A (Agilent Technologies, Inc.), and the signal was recovered from the friction sensor with the use of a signal-conditioning amplifier Vishay, Measurements (2310B).^40^ The sample preparation protocol is described in Section 2.4.

### 2.3 Preparation of Bottlebrush Polymers

#### 2.3.1 Preparation of Monoblock poly(2-(2-bromoisobutyryloxy)ethyl methacrylate-*co*-methyl methacrylate), P(BIBEMA-*co*-MMA), by RAFT Polymerization

CPBDT (11.06 mg, 0.05 mmol), MMA (3.50 g, 35.0 mmol), BIBEMA (9.82 g, 35.0 mmol), and anisole (5 mL) were charged in a dry 25 mL Schlenk flask. Then, AIBN as a reaction initiator (1.64 mg, 0.01 mmol) was taken from a stock solution in DMF and added to the reaction flask. The mixture was deoxygenated by argon gas bubbling for approximately 30 min. Afterward, the polymerization was initiated by immersing the flask in a preheated oil bath at 70 °C. To monitor the progress of the polymerization, at predetermined time points, an aliquot of the sample was taken from the reaction mixture under an inert atmosphere and analyzed by ^1^H NMR spectroscopy. The reaction was terminated by exposing the reaction mixture to air at a desired monomer conversion. Finally, the reaction mixture was precipitated three times in cold methanol to obtain a pure polymer. The resulting polymer was dried under vacuum at 30 °C overnight. The obtained bottlebrushes were used as a reference system for further studies.

#### 2.3.2 General Procedure for the Synthesis of Monoblock poly(methyl methacrylate-*co*-monomer X)-CTA, P(MMA-*co*-X)-CTA, by RAFT Polymerization

First, CPBDT (0.1 mmol) was charged in a dry Schlenk flask. Then, MMA (15 mmol), monomer X (GMA, tBMA, or FBMA) (15 mmol), and DMF (2 mL) were also injected into the Schlenk flask. AIBN as an initiator (0.015 mmol, taken from a stock solution of DMF) was added to the flask and deoxygenated via argon flux for 30 min. Subsequently, the polymerization was initiated by immersing the flask in a preheated oil bath at 70 °C. The polymer obtained was precipitated three times in cold methanol to obtain the pure polymer and then dried under vacuum at 30 °C overnight. The obtained copolymer was the anchoring block (cartilage-binding block) in final materials.

#### 2.3.3 General Procedure for the Synthesis of Poly(methyl methacrylate-*co*-monomer X)-*b*-poly(2-(2-bromoisobutyryloxy)ethyl methacrylate-*co*-methyl methacrylate), P(MMA-*co*-X)-*b*-P(BIBEMA-*co*-MMA), by RAFT Polymerization

P(MMA-*co*-X)-CTA (0.1 mmol) was placed in a dry Schlenk flask and dissolved in DMF (2 mL). Then, MMA (50 mmol), BIBEMA (50 mmol), and AIBN as an initiator (0.015 mmol, taken from a stock solution of DMF) were injected into the flask. The flask was deoxygenated for 30 min and subsequently immersed in a preheated oil bath at 70 °C to initiate the polymerization. For purification, the obtained polymer was precipitated three times in cold methanol and the resulting precipitate was dried under vacuum at 30 °C overnight. The obtained copolymer is the anchoring block extended by the macroinitiator block for side chain synthesis.

#### 2.3.4 General Procedure for the Synthesis of Poly(monomer X-*co*-methyl methacrylate)-*b*-(poly(2-(2-bromoisobutyryloxy)ethyl methacrylate-*co*-methyl methacrylate)-*g*-poly(2-methacryloyloxyethyl phosphorylcholine)) Bottlebrush Polymers, P(MMA-*co*-X)-*b*-(P(BIBEMA-*co*-MMA)-*g*-PMPC), by ATRP Polymerization

A dry 5 mL Schlenk flask was charged with P(BIBEMA-*co*-MMA) or P(MMA-*co*-X)-*b*-P(BIBEMA-*co*-MMA) as macroinitiators (0.018 mmol), MPC (2.25 mmol), bpy (0.0612 mmol), and copper(II) chloride (0.0036 mmol). The flask was degassed by using a vacuum pump and then flowing argon gas. Afterward, deoxygenized methanol (2.5 mL) and acetonitrile (2.5 mL) were added to the flask to dissolve the mixture. The mixture solution was again deoxygenated by three freeze–pump–thaw cycles. During the final cycle, the flask was filled with argon, and CuCl (0.027 mmol) was quickly added to the frozen reaction mixture. The flask was sealed, degassed, and backfilled with argon five times and then immersed in an oil bath at 60 °C. The reaction was stopped after reaching the desired monomer conversions, by exposing to air. The resulting bottlebrush polymers were purified by dialysis (pore size molar mass cut off 50,000 Da) against methanol for 4 days to remove all unreacted monomers and copper catalysts. Before the final dialysis, bottlebrush polymer with *tert*-butyl methacrylate units was dissolved in methanol/acetonitrile mixture containing trifluoroacetic acid and stirred overnight. Finally, the polymer solution was also dialyzed against deionized water for an additional 3 days to obtain bottlebrush polymers in an aqueous solution. The resulting solution was freeze-dried, and the obtained powders were stored under an inert atmosphere and used for further studies.

### 2.4 Lubrication Tests on the Mica Surface

#### 2.4.1 Mica Preparation

The mica surfaces for the lubrication experiments were prepared as previously reported.^41,42^ The freshly cleaved transparent mica was coated by physical vapor deposition with a 55 nm-thick silver layer. Pieces with the same thickness were glued with silver epoxy glue on cylindrical glass discs with a curvature of 2 cm.

#### 2.4.2 Surface Modification

The mica surfaces were modified with amino groups using (3-aminopropyl)triethoxysilane (APTES) as described previously.^43,44^ The mica surfaces were activated in a plasma chamber under a vacuum pressure of 0.5 mTorr. Gaseous argon and water vapor were introduced into the chamber at a partial pressure of 60 mTorr and 300 mTorr, respectively. Plasma activation was performed for 10 min and after that, the surfaces were left under vacuum for 5 min. Then, the activated mica surfaces were transferred to a desiccator with a small reservoir containing 100 μL of APTES and incubated for 3 h under vacuum at a pressure of 1.6 mmHg at room temperature. The mica surfaces were then thoroughly rinsed with pure ethanol. To complete the APTES covalent surface grafting, annealing was carried out at 120 °C under atmospheric pressure for 30 min. The modified surfaces were placed in the SFA chamber and exposed to air to measure the thickness of the APTES layer.

#### 2.4.3 Lubrication Test

The lubrication tests were performed on both neat and modified mica pieces. Bottlebrush aqueous solutions (40 μg/mL) were injected between two mica surfaces and kept for 1 h to allow polymer adsorption. To reduce the effects of water evaporation, a small reservoir with water was placed in the SFA chamber. Before each shear cycle, the normal force was set and monitored using semiconductor strain gauges mounted on the double cantilever of the bimorph slider. For each applied load (ranging from low to high), the bimorph drove the lower surface in a back-and-forth motion controlled by a function generator (Agilent 33250A, Agilent Technologies, Inc.) using a triangular wave function with a typical frequency of 50 mHz and an amplitude of 5 V (corresponding to 5 μm/s and 50 μm). During sliding, frictional forces transmitted to the upper surface were measured by two vertical double-cantilever springs, each equipped with four semiconductor strain gauges. These gauges were attached symmetrically to oppositely bending arms of the springs, forming the four arms of a Wheatstone bridge strain gauge system. When a lateral force was applied to the upper surface, the strain gauges measured the deflection, and a signal-conditioning amplifier (Vishay Measurements, 2310B) outputted the signal to a computer data acquisition recorder (Soltec TA220-2300A). The friction force was measured during the experiment at different normal loads up to 5 mN (maximum contact pressure of 10 MPa). At least three cycles were measured and analyzed at each applied load.

### 2.5 Lubrication Tests on Articular Cartilage Tissue

#### 2.5.1 Articular Cartilage Sample Preparation

The chicken knee joints were exposed with a scalpel. The joints were evaluated in terms of cartilage tissue based on the color and visible damage on the surface. Only white areas with no mechanical damage were collected. The lower part of the cartilage attached to the bone was cut completely flat to place on the smooth surface of the disc. The cartilage could have a round or nearly flat shape. Collected cartilage pieces were kept in Milli-Q water for 1 h to rinse them from water-soluble contaminants. The cartilage was transferred to the PBS solution and stored at 4 °C until further use. Before the experiment, a couple of cartilages were cut side down on dust-free wipe to soak part of the liquid and after 10 s glued to flat glass discs using waterproof glue. The waterproof glue used is Sky Eyelash Glue, with each drop measuring 30 μL. Only one drop of glue was used to attach the cartilage to the disk, ensuring that the cartilage remained thick enough to retain its original mechanical properties.

#### 2.5.2 Lubrication Tests

To mitigate the variability in stiffness and roughness from sample to sample, the initial friction test for each cartilage pair was conducted with a PBS solution interposed between the surfaces. The friction tests were performed at several normal loads ranging from 0 to 8 mN (up to a maximum contact pressure of 200 kPa) using the same experimental parameters used with the mica surfaces described in Section 2.4.3. The shearing speed was between 3 and 4 μm/s. Because of the specificity of the cartilage samples (highly opaque material, compliant, porous and rough), the contact area could not be quantified based on FECO analysis. For this reason, the contact area was estimated using the Hertz model and based on the normal load applied (and recorded through the strain gauges attached to the normal double cantilever), the radius of the cartilage samples (1.1–2.9 mm), and the compression modulus of the cartilage (10 MPa).

After testing lubrication in PBS, the surfaces were separated, and the PBS solution was removed with a syringe and gently washed by adding and removing water three times. Then, a solution of polymer (40 μg/mL) in *mili-Q* water was injected and left to adsorb for 1 h. The lubrication tests were performed under the same conditions as described for PBS solution alone. The friction test was resumed with the injected sample, and the obtained frictional data were compared with those acquired using PBS for the same cartilage pair.

## 3 Results and Discussion

### 3.1 Synthesis and Characterization of Bottlebrush Polymers

**Scheme 1:**
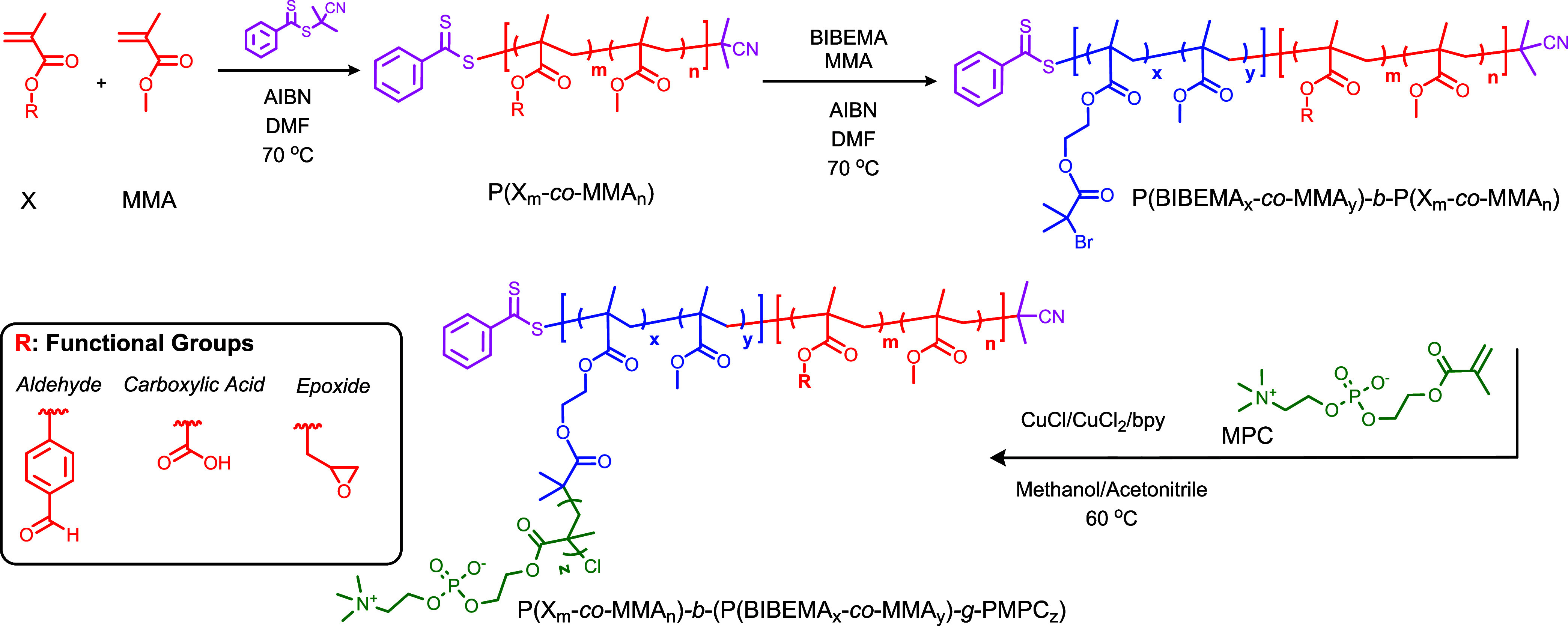
Synthetic Procedure for the Preparation of Bottlebrush Polymers with Anchoring Groups. *Step 1*: Copolymerization of MMA and Monomer X for the Synthesis of Monoblock P(X-*co*-MMA) via RAFT Polymerization. *Step 2*: Synthesis of a Diblock Copolymer of P(X-*co*-MMA)-*b*-P(BIBEMA-*co*-MMA) via RAFT Polymerization. *Step 3*: Grafting of MPC on the Polymeric Backbone for the Synthesis of the P(X-*co*-MMA)-*b*-(P(BIBEMA-*co*-MMA)-*g*-PMPC) Bottlebrush via ATRP

In this study, a series of bottlebrush polymers with different functional groups including aldehyde, carboxylic, and epoxide at the anchoring side (cartilage-binding side) were designed and synthesized as shown in Scheme 1. Two reversible-deactivation radical polymerization (RDRP) techniques, atom transfer radical polymerization (ATRP) and reversible addition–fragmentation chain transfer polymerization (RAFT), were selected for the synthesis of the bottlebrush polymers. These methods provide good control over the molecular weight of the synthesized polymers and enable the synthesis of brushes with different compositions and the functionality of side chains. The molecular structure of the synthesized bottlebrushes was identified by FTIR, ^1^H NMR, SEC, and DLS analysis. Preliminary verification of the application potential of the materials obtained as lubricants that mimic the natural lubricin was done based on the friction profiles. To compare the anchoring properties of different functional groups, the friction tests were performed on neat mica and amine-functionalized mica (surface modification by APTES).

#### 3.1.1 Cartilage Binding Block

Considering the nature of the functional groups present on the cartilage surface and further understanding the influence of the interactions between the cartilage surface and synthesized bottlebrush polymers, three functional monomers were chosen to form the first anchoring block of brush macromolecules. Thus, 4-formylphenyl methacrylate (FPMA), *tert*-butyl methacrylate (*t*BMA), and glycidyl methacrylate (GMA) were separately copolymerized with MMA by RAFT polymerization. RAFT polymerization was performed in DMF as a solvent at 70 °C using CPBDT and AIBN as a RAFT chain transfer agent and initiator, respectively. To obtain the first block with a degree of polymerization of around 100, the molar ratio of 1.0:150:150:0.15 for CPBDT:monomer X: MMA:AIBN was used.

To determine the chemical structure of the synthesized anchoring blocks, ^1^H NMR spectroscopy was performed for all samples (Figure S3). In the ^1^H NMR spectrum of the P(FBMA_59_-*co*-MMA_49_) copolymer with the aldehyde pending group, two characteristic signals at 10.01 ppm and 7.90–7.93 ppm were assigned to the aldehyde group (e, O=C–H) and aromatic ring (d, C–H), respectively. Furthermore, peaks related to the methyl and methylene groups (a, b, −CH_3_–, −CH_2_−) are visible between 1.08 and 2.09 ppm (Figure S3a). In the ^1^H NMR spectrum of the P(*t*BMA_50_*-co-*MMA_48_) anchoring block, there is a main characteristic signal of the *tert*-butyl group (−OC(CH_3_)_3_) at 1.45 ppm (Figure S3b). The ^1^H NMR spectrum of P(GMA_49_-*co*-MMA_46_) showed the characteristic peaks of the pending epoxide groups at 2.64 and 2.86 ppm for the methylene (−CH_2_) of the epoxide ring, 3.23 ppm for the methine (−CH−) of the epoxied ring, and 3.83 and 4.29 ppm for the methylene groups (C–CH_2_CH_2_–O) (Figure S3c). Peaks related to the methyl group, −OCH_3_, of the MMA units in all anchoring blocks can be observed in the range of 3.50–3.65 ppm.

The FTIR spectra of anchoring blocks, P(FBMA_59_-*co*-MMA_49_), P(*t*BMA_50_-*co*-MMA_48_), and P(GMA_49_-*co*-MMA_46_), are shown in Figure S4. They are typical for polymethacrylates and contain the characteristic set of spectral lines for this class of compounds located in the following ranges: 2850–3000 cm^–1^ (assigned to the C–H stretching of aliphatic chains), 1718 with a shoulder at 1730 cm^–1^ (C=O stretching of the ester group), 1420–1520 cm^–1^ (bending of the CH_2_ and CH_3_ groups), and 1238 and 1161 cm^–1^ (symmetric and asymmetric stretching of the C–O–C group, respectively).^45^ Moreover, for the P(FBMA_59_-*co*-MMA_49_) copolymer, line characteristic for the stretching of C=O of the aldehyde group is visible at c.a. 1700 cm^–1^. Furthermore, the characteristic band for the aromatic ring occurs at c.a. 1600 cm^–1^ (C=C stretching in aromatic rings).^46^ For P(GMA_49_-*co*-MMA_46_) copolymer, the line characteristic for the epoxide ring is visible at c.a. 910 cm^–1^.^47,48^ Most of the vibrational bands of the *tert*-butyl group overlap with lines of MMA; the spectrum of the P(*t*BMA_50_-*co*-MMA_48_) copolymer is similar to the spectrum of a similar copolymer (PMMA-*b*-P*n*BMA) reported previously.^45^

SEC analysis was performed to characterize the molecular weight and dispersity of synthesized anchoring blocks (Figure S5). For all the anchoring block copolymers, a single symmetric peak in the SEC chromatograms had a monomodal distribution of molecular weight. The dispersity (*M*_w_/*M*_n_) of the obtained copolymers was 1.48, 1.24, and 1.41 for the anchoring blocks P(FBMA_59_-*co*-MMA_49_), P(*t*BMA_50_-*co*-MMA_48_), and P(GMA_49_-*co*-MMA_46_), respectively. These results confirm the well-defined structure of the copolymers prepared by RAFT polymerization. Based on SEC result analysis, the relative molecular weights (*M*_n_) for all anchoring block polymers were determined to be around 13,200, 11,600, and 9000 g/mol for P(FBMA_59_-*co*-MMA_49_), P(*t*BMA_50_-*co*-MMA_48_), and P(GMA_49_-*co*-MMA_46_), respectively, which were lower than the theoretical value (*M*_n theor_) obtained based on ^1^H NMR spectroscopy results (16,300, 12,100, and 12,100 g/mol, respectively).

#### 3.1.2 Lubricating Block: Synthesis of the Bottlebrush Backbone

To synthesize the second block of the bottlebrush, as a lubricating block, the predried anchoring block copolymers were extended by RAFT polymerization to obtain P(X-*co*-MMA)-*b*-P(BIBEMA-*co*-MMA) block copolymers using a molar ratio of macroinitiator:BIBEMA:MMA:AIBN = 1.0:500:500:0.15. Because the added block is considered the backbone of the final bottlebrush polymer, its length was chosen to be 5 times higher than the length of the anchoring block.

**Figure 2:**
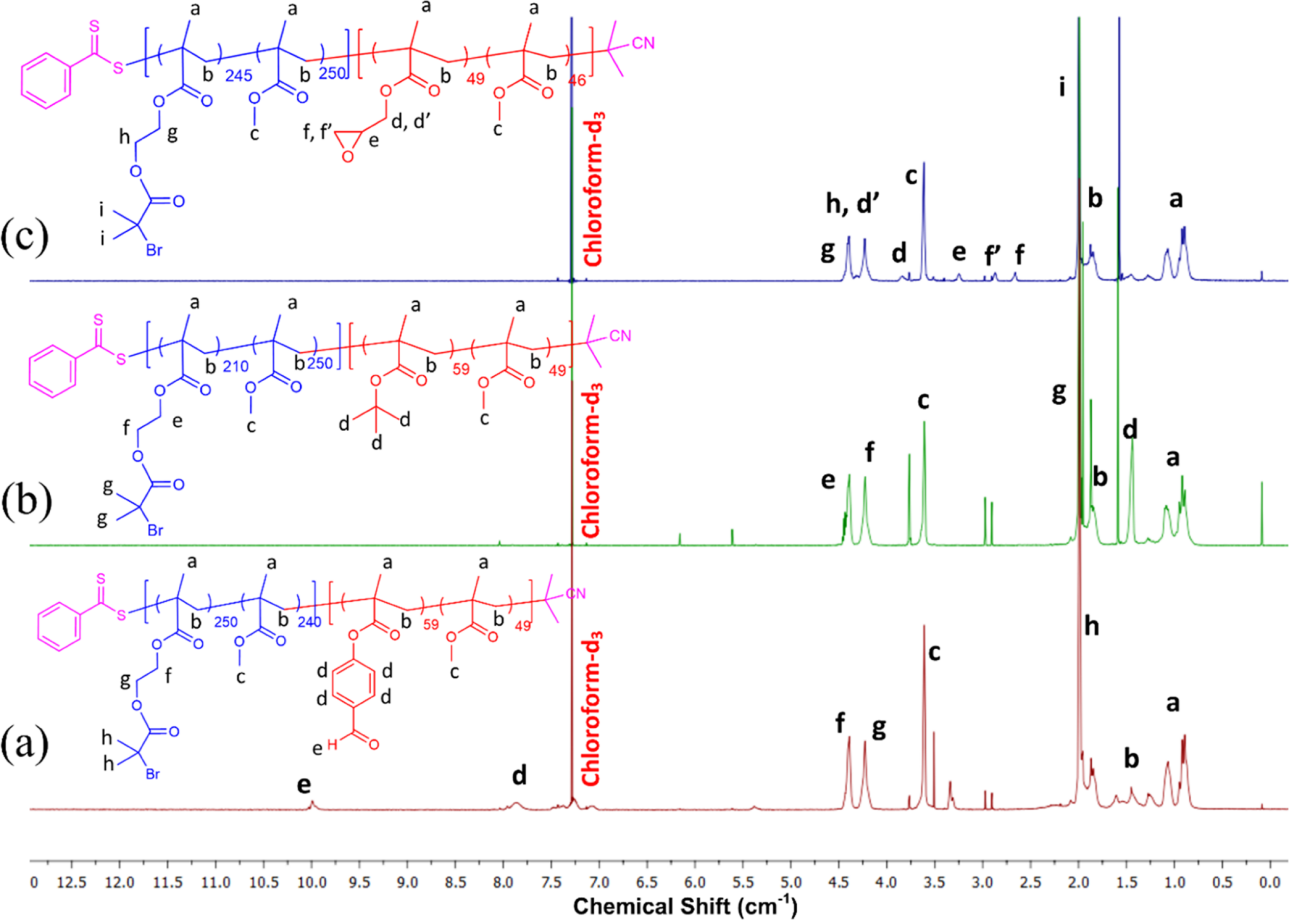
^1^H NMR spectra of P(FBMA_59_-*co*-MMA_49_)-*b*-P(BIBEMA_250_-*co*-MMA_240_) (a), P(*t*BMA_50_-*co*-MMA_48_)-_b_-P(BIBEMA_210_-*co*-MMA_250_) (b), and P(GMA_49_-*co*-MMA_46_)-*b*-P(BIBEMA_245_-*co*-MMA_250_) (c) in chloroform-d_3_.

The ^1^H NMR spectrum of P(FBMA_59_-*co*-MMA_49_)-*b*-P(BIBEMA_250_-*co*-MMA_240_) showed the peaks characteristic for the aldehyde group and C–H aromatic ring, which appeared at 9.99 and 7.85 ppm, respectively. They are slightly shifted in relation to the peaks observed for P(FPMA_59_-*co*-MMA_49_). Both peaks showed low intensity because of the extension of the initial copolymer with a long polymeric chain. The other characteristic peaks for the P(BIBEMA_250_-*co*-MMA_240_) block appeared at 1.95 and 3.61 ppm and could be assigned to the dimethyl groups of BIBEMA and the methyl groups (−OCH_3_) of MMA, respectively (Figure 2a). Regarding the P(*t*BMA_50_-*co*-MMA_48_)-*b*-P(BIBEMA_210_-*co*-MMA_250_), the characteristic peak related to the methoxy, −OCH_3_, and *tert*-butoxy, -OC(CH_3_)_3_, groups, appeared at 3.5 and 1.45 ppm, respectively. Furthermore, the peak related to the methylene (−OCH_2_CH_2_O−) and dimethyl groups of BIBEMA could be observed at 4.5, 4.25, and 1.8 ppm (Figure 2b). The ^1^H NMR spectrum of P(GMA_49_-*co*-MMA_46_) demonstrated the characteristic peaks of epoxide pending groups at 2.64 and 2.86 ppm for methylene (−CH_2_−) of the epoxide ring, 3.23 ppm for the methylene (−CH−) of the epoxide ring, and 3.83 and 4.29 ppm for methylene groups (C–CH_2_CH_2_–O), (Figure 2c).

The FTIR spectra of the copolymers that contain both the anchoring block and the backbone of the lubricating block are shown in Figure S6. They are dominated by the bands characteristic for methyl methacrylate monomer units, as this is the predominant comonomer in each copolymer. The most important is the presence of the lines characteristics for functional (anchoring) groups. For the P(GMA_49_-*co*-MMA_46_)-*b*-P(BIBEMA_245_-*co*-MMA_250_) copolymer, the line characteristic for the epoxide ring is visible after deep analysis as a weak band at c.a. 910 cm^–1^. For the P(FBMA_59_-*co*-MMA_49_)-*b*-P(BIBEMA_250_-*co*-MMA_240_) copolymer, a band characteristic for stretching of C=C bonds in the phenyl ring is present, and additionally, the band related to C=O stretching is significantly broadened in comparison to other bottlebrush systems. This confirms the presence of aldehyde groups in this material. In the FTIR spectrum of P(*t*BMA_50_-*co*-MMA_48_)-*b*-P(BIBEMA_210_-*co*-MMA_250_), there are no separate band characteristics for the *tert*-butyl group. In general, the FTIR spectra are consistent with the ^1^H NMR spectra and proved that the anchoring groups survived the next step of the synthesis.

**Figure 3:**
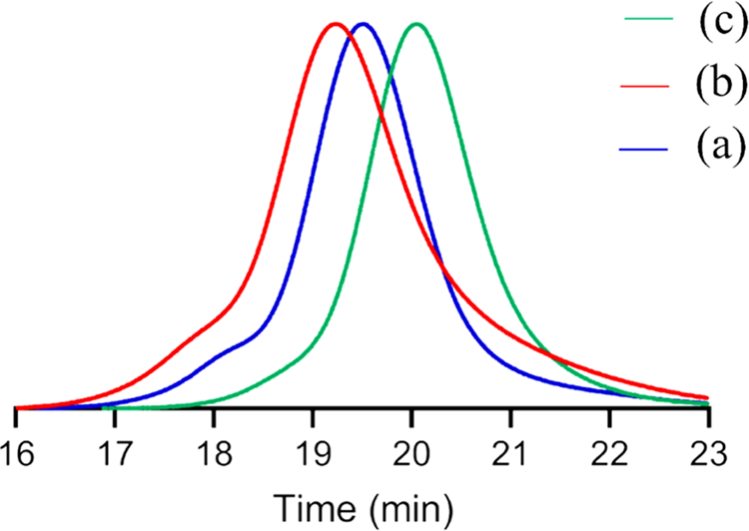
SEC chromatograms of P(FBMA_59_-*co*-MMA_49_)-*b*-P(BIBEMA_250_-*co*-MMA_240_) (a), P(*t*BMA_50_-*co*-MMA_48_)-*b*-P(BIBEMA_210_-*co*-MMA_250_) (b), and P(GMA_49_-*co*-MMA_46_)-*b*-P(BIBEMA_245_-*co*-MMA_250_) (c).

To characterize the molecular weight and dispersity of the synthesized P(FBMA_59_-*co*-MMA_49_)-*b*-P(BIBEMA_250_-*co*-MMA_240_), P(*t*BMA_50_-*co*-MMA_48_)-*b*-P(BIBEMA_210_-*co*-MMA_250_), and P(GMA_49_-*co*-MMA_46_)-*b*-P(BIBEMA_245_-*co*-MMA_250_), SEC analysis was performed. All polymers showed a single peak with a small shoulder within high molecular weight in the case of the synthesized P(FBMA_59_-*co*-MMA_49_)-*b*-P(BIBEMA_250_-*co*-MMA_240_), and P(GMA_49_-*co*-MMA_46_)-*b*-P(BIBEMA_245_-*co*-MMA_250_). The apparent molecular weights based on linear PMMA standards of the polymers were *M*_n_ = 48,900, 41,100, and 53,100 g/mol, respectively, which were lower than the theoretical value obtained based on the results of the ^1^H NMR spectroscopy, *M*_n theor_ = 109,900, 95,500, and 105,200 g/mol (Table 1). In addition, for all copolymers, low dispersity values were found (*M*_w_/*M*_n_ = 1.5, 1.18, and 1.25, respectively). These results indicate that the structures of the copolymers prepared by RAFT polymerization were well-defined; regardless, a small contribution of radical coupling was visible (Figure 3).

**Table 1:** Comparison of SEC and ^1^H NMR Results for Monoblock and Diblock Copolymers

	monoblock composition (DP)^a^		diblock composition (DP)^a^				
sample	X^c^	MMA	BIBEMA	MMA	*M*_n theor_^a^ g/mol	*M*_n_^b^ g/mol	*Đ*^b^
P(FBMA-*co*-MMA)	59	49			16,300	13,200	1.48
P(*t*BMA-*co*-MMA)	50	48			12,100	11,600	1.24
P(GMA-*co*-MMA)	49	46			12,100	9,000	1.41
P(BIBEMA-*co*-MMA)^d^			246	249	93,500	53,400	1.28
(PFBMA-*co*-MMA)-*b*-P(BIBEMA-*co*-MMA)	59	49	250	240	109,900	48,900	1.50
P(*t*BMA-*co*-MMA)-*b*-P(BIBEMA-*co*-MMA)	50	48	210	250	95,500	41,100	1.18
P(GMA-*co*-MMA)-*b*-P(BIBEMA-*co*-MMA)	49	46	245	250	105,200	53,100	1.25

a Theoretical molecular weight and degree of polymerization based on monomer conversion calculated from 1H NMR spectroscopy results.

b *M*_n_ and *Đ* determined by SEC using linear PMMA standards in DMF.

c X: *t*BMA, GMA, and FBMA.

d Reference polymer (monoblock) for bottlebrushes without an anchoring block, Figure S9–S11.

#### 3.1.3 Side Chains of the Bottlebrush Block

The side chains of the brush block of the designed polymer were composed of methacrylate-type polymeric chains with zwitterionic branches of poly(2-methacryloyloxyethyl phosphorylcholine) (PMPC). The use of PMPC as side chains was expected to enhance the biocompatibility of the final bottlebrush polymers as well as to exhibit extremely good lubrication under physiological conditions.

**Figure 4:**
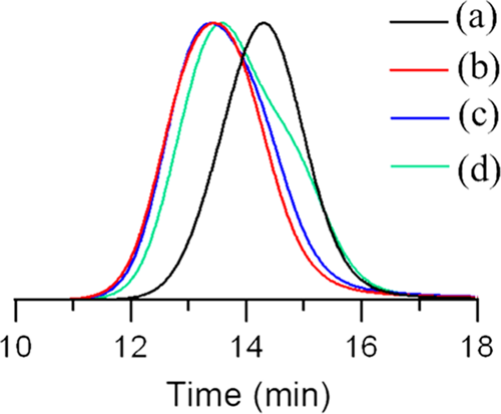
SEC-MALS results for bottlebrush polymers P(BIBEMA_246_-*co*-MMA_249_)-*g*-PMPC_52_ (a), P(FBMA_59_-*co*-MMA_49_)-*b*-(P(BIBEMA_250_-*co*-MMA_240_)-*g*-PMPC_48_) (b), P(MAA_50_-*co*-MMA_48_)-*b*-(P(BIBEMA_210_-*co*-MMA_250_)-*g*-PMPC_61_) (c), and P(GMA_49_-*co*-MMA_46_)-*b*-(P(BIBEMA_245_-*co*-MMA_250_)-*g*-PMPC_50_) (d) using PBS buffer (pH 7.4) and 100 mM sodium phosphate (pH 2.5) with 0.2 vol % of trifluoroacetic acid as an eluent.

The ^1^H NMR spectrum of bottlebrush polymers showed characteristic resonance signals of protons related to methyl groups (a, −CH_3_) at δ = 1.90–1.98 ppm and methylene groups (b, f, −CH-C−) at δ = 0.9–1.4 ppm. All methylene protons (i, j, k, l, −OCH_2_CH_2_O−) related to the PMPC side chain were observed at δ = 4.36, 4.25, 4.11, and 3.79 ppm that overlapped with the methylene groups (d, e, −OCH_2_CH_2_O−) of the lubricating block. The signals of methyl protons connected to the terminal quaternary amine (N^+^–CH_3_) that appeared at δ = 3.33 ppm. The aldehyde, carboxylic, and epoxide functional groups from the anchoring block were no longer visible due to the low molar ratio of the cartilage-binding bock compared to the entire copolymer (weight ratio: ∼0.002) (Figure S7). The SEC-MALS analysis showed that all the bottlebrush polymers were uniform as SEC peaks were symmetric. Also, low dispersity for all obtained bottlebrush polymers was found, except the brush with epoxide functional groups in which slightly broader molecular weight distribution was detected (Figure 4). Average molecular weights (*M*_n_ and *M*_w_), dispersity (*Đ*), radius of gyration (*R*_g_), and radius of a theoretical sphere (*R*_h_) are reported for all bottlebrush polymers in Table 2. The bottlebrush without any anchoring block was also synthesized as a reference system. The polymerization conditions and detailed brush characterization data are given in the SI (Scheme S1, Figures S6–S9).

**Table 2:** SEC-MALS and DLS Results of Bottlebrush Polymers

				**SEC-MALS**				
Bottlebrush polymers	*d*_n_/*d*_c_^a^ (mL/g)	*M*_n th_. (kg/mol)	*size*, *PDI*_(nm)_	*M*_n_^b^ (kg/mol)	*M*_w_^b^ (kg/mol)	*Đ*^b^_(*M*_w_/*M*_n_)_	*R*_g (nm)_	*R*_h (nm)_
**P(BIBEMA**_**246**_**-*****co*****-MMA**_**249**_**)-*****g*****-PMPC**_**52**_^c^	0.126	4700	53 nm, 0.316	3632	3942	1.09	28.2	38.2
**P(FBMA**_**59**_**-*****co*****-MMA**_**49**_**)-*****b*****-(P(BIBEMA**_**250**_**-*****co*****-MMA**_**240**_**)-*****g*****-PMPC**_**48**_**)**	0.126	4870	65 nm, 0.136	11,786	14,019	1.19	59.7	57.6
**P(MAA**_**50**_**-*****co*****-MMA**_**48**_**)-*****b*****-(P(BIBEMA**_**210**_**-*****co*****-MMA**_**250**_**)-*****g*****-PMPC**_**61**_**)**	0.123	4440	31 nm, 0.289	10,328	12,995	1.26	52.7	57.5
**P(GMA**_**49**_**-*****co*****-MMA**_**46**_**)-*****b*****-(P(BIBEMA**_**245**_**-*****co*****-MMA**_**250**_**)-*****g*****-PMPC**_**50**_**)**	0.130	4450	44 nm, 0.286	3787	5667	1.50	45.8	54.8

a Measured by manual injection onto the RI detector of the samples with varying concentrations in PBS.

b Absolute molar masses (*M*_n_ and *M*_w_) and dispersity (*Đ*) were determined by SEC analysis (DPBS as eluent) with MALS detectors using PBS as an eluent. *M*_n_ and *M*_w_ were calculated with measured d*n*/d*c* values of each polymer.

c Reference bottlebrush without anchoring an block, Figure S9–S12.

In the FTIR spectrum of the bottlebrush polymer containing the PMPC block, three lines characteristic for the vibrations of the −PO–CH_2_– groups, namely 1234, 1152, and 1076 cm^–1^, were observed (Figure S8). Additionally, the broad band in the range of 3200–3400 cm^–1^ confirmed the presence of hydroxyl groups.^49^ The lines typical for quaternary amines are visible at 720 cm^–1^ (C–N stretching), 920–930 cm^–1^ (stretching C–N^+^(CH_3_)_3_), and 950 cm^–1^ (bending N^+^(CH_3_)_3_).^50^ All these lines confirm that the phosphorylcholine groups were successfully introduced to the structure of the bottlebrush polymers.

**Figure 5:**
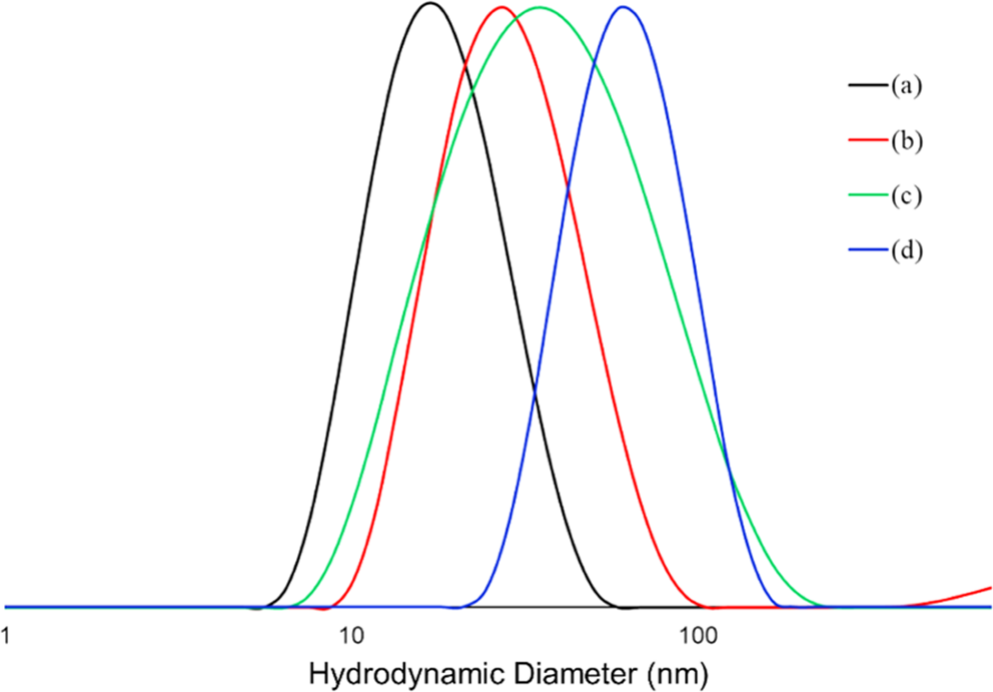
Hydrodynamic diameter (*D*_h_) of P(BIBEMA_246_-*co*-MMA_249_)-*g*-PMPC_52_ (**a**), PGMA_49_-*co*-PMMA_46_-*b*-PBIBEMA_245_-*g*-PMPC_50_-*co*-PMMA_250_ (**b**), PMAA_50_-*co*-PMMA_48_-*b*-PBIBEMA_210_-*g*-PMPC_61_-*co*-PMMA_250_ (**c**), and PFBMA_59_-*co*-PMMA_49_-*b*-PBIBEMA_250_-*g*-PMPC_48_-*co*-PMMA_240_ (**d**) determined by dynamic light scattering in water at 25 °C.

The dynamic light scattering measurements, DLS, were performed to measure the hydrodynamic diameter (*D*_h_) of the polymers in water at a concentration of 5 mg/mL. As seen in Figure 5, all polymers showed very low hydrodynamic diameter in the range of 30–65 nm with a narrow size distribution. The results of DLS are very consistent with the results obtained from the SEC-MALS. Furthermore, the particle size distribution described by PDI for all bottlebrush polymers with anchoring blocks was in the range of 0.135–0.290, which confirmed the relatively narrow distribution of the size of bottlebrush polymers in the aqueous solution.

**Figure 6:**
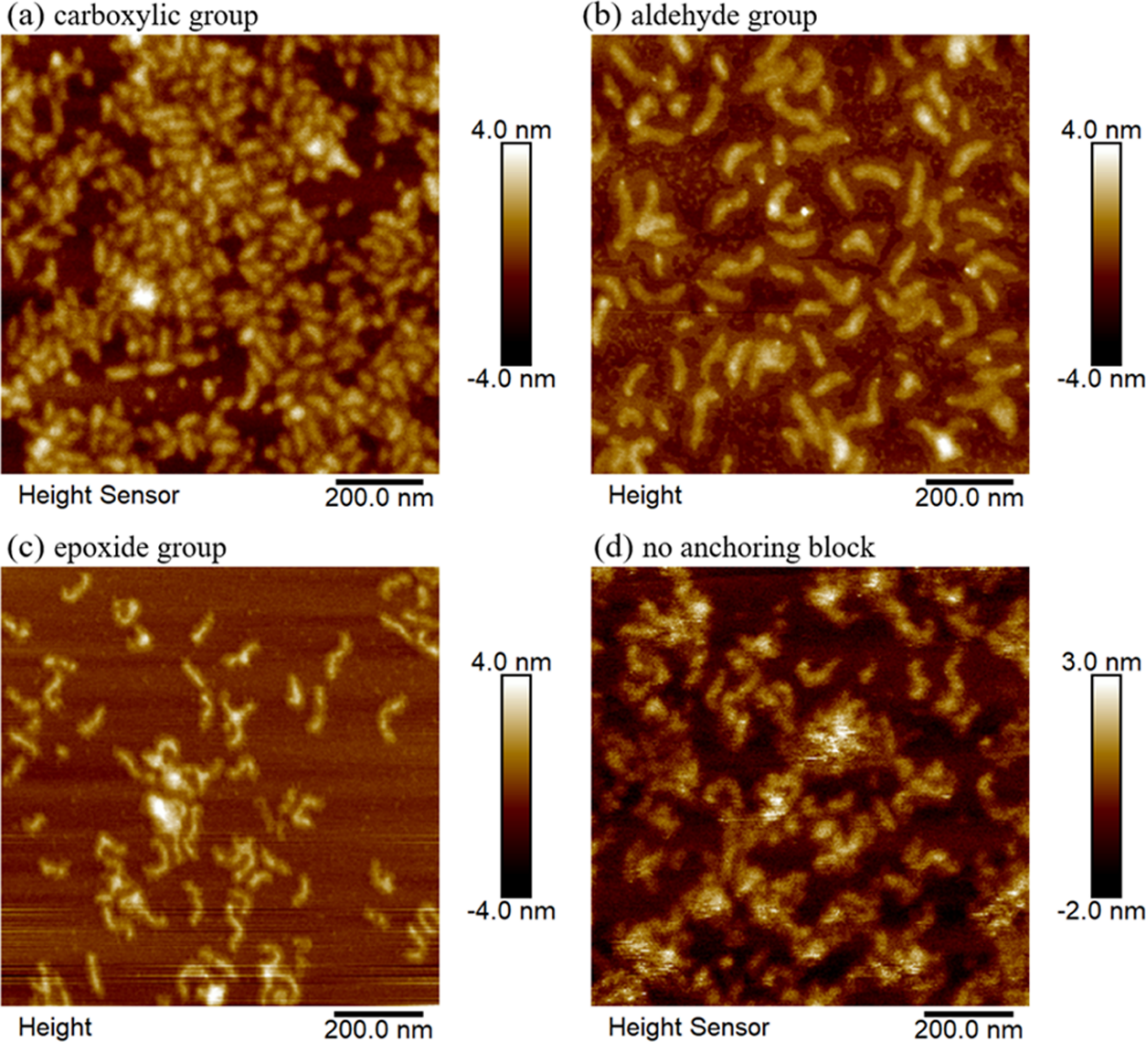
Atomic force microscopy images of bottlebrush polymers with different binding blocks: carboxylic (a), aldehyde (b), epoxide (c), and no anchoring group (d).

The atomic force microscopy (AFM) images (Figure 6) confirmed the finely dispersed nature of polymeric chains in water after their deposition on mica surfaces. The bottlebrush polymers exhibited a characteristic elongated shape and displayed low aggregation content. Contour lengths of bottlebrush polymer chains were calculated in ImageJ software based on AFM images: for the monoblock polymers, the contour length was 72.4 ± 7.1 nm, for the diblock with a carboxylic group, it was 64.8 ± 6.4 nm, for those with an aldehyde group, it was 104.9 ± 9.2 nm, and for those with an epoxide group, it was 77.5 ± 11.4 nm. The observed differences in the contour length of the bottlebrush polymers anchored with various functional groups can be related to the slightly different degree of polymerization of particular blocks, specifically backbones and side chains of each system (refer to Table 2). Those were the highest for aldehyde-containing brushes. Also, the size and shape of the anchoring groups (aldehyde, carboxylic acid, and epoxide) could influence how efficiently the polymer chains pack on the surface. Groups like aldehydes might promote intermolecular interactions between single brushes, leading to a higher average contour length (104.9 nm) compared to the more compact molecules with carboxylic and epoxide groups (64.8 and 77.5 nm, respectively).

### 3.2 Lubrication Tests

#### 3.2.1 Anchoring on Mica Surfaces

The aldehyde, carboxylic, or epoxide anchoring groups within bottlebrush polymers should provide strong interactions with the amine and hydroxy groups present on most biological surfaces such as cartilage. All these anchoring functional groups can interact via hydrogen bonding and electrostatics or even form covalent bonds with surfaces exhibiting either hydroxy or amine (primary) functions. To demonstrate this versatility, the lubricating properties of different polymers on mica were tested with the use of SFA equipment. Details of the experiment can be found in Section 2. The mica surface exhibits a high density of hydroxyl functional groups, which are chemically stable.^51^ Mica was also modified with APTES to introduce covalently grafted primary amine groups on the surface. A bottlebrush copolymer without any anchoring group was used as a control condition to measure the impact of the chemical nature of the anchoring group on the lubricating properties of the polymers. To compare the effectiveness of the different anchoring groups, each bottlebrush polymer was measured on both nonmodified and modified mica surfaces in at least three separate experiments. For each experimental condition, a set of 2–3 pairs of mica surfaces were tested independently. For each pair of surfaces, lubrication tests were performed on 1–3 distinct contact points.

**Figure 7:**
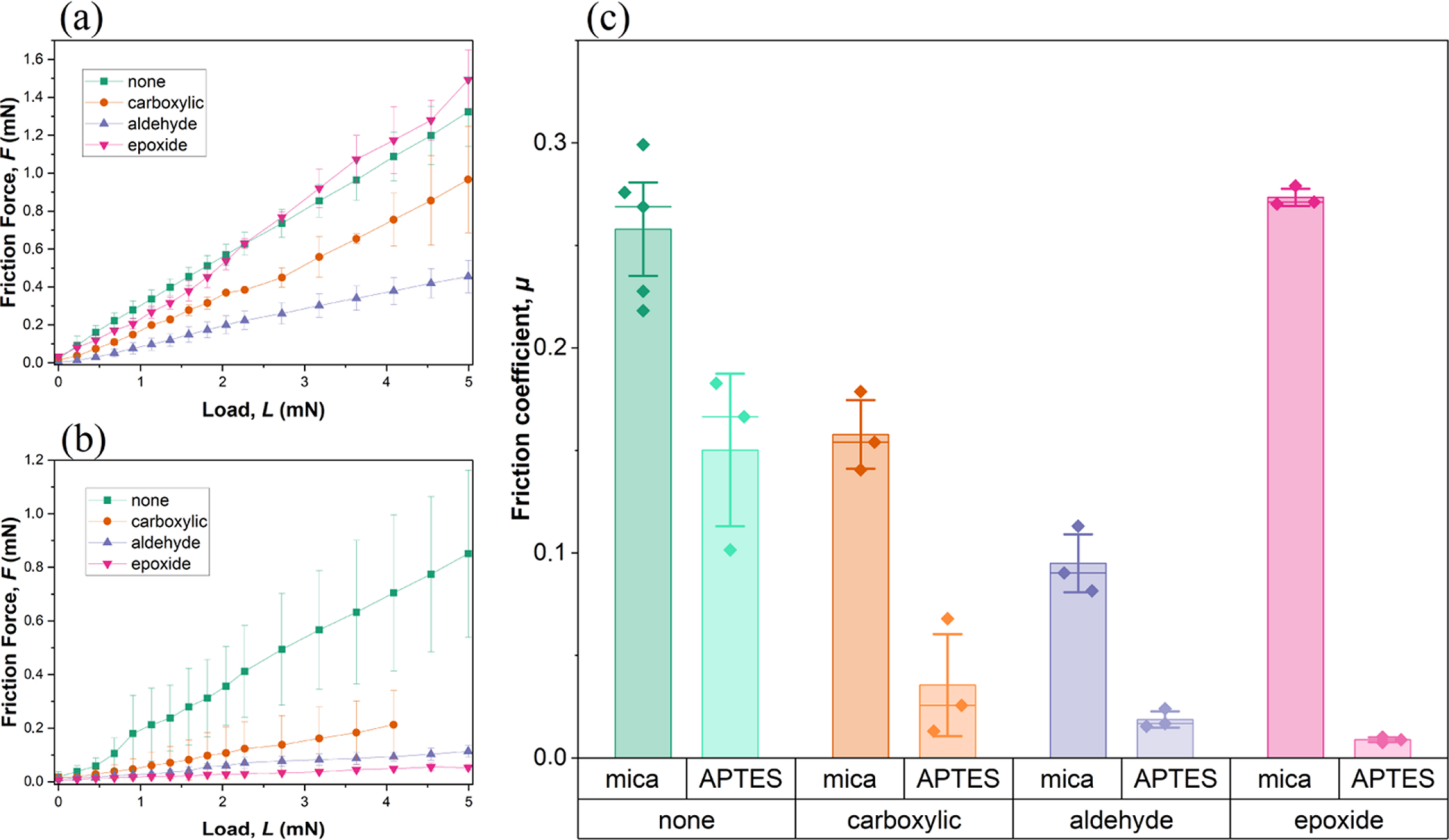
Results of dynamic friction force measurements performed on mica surfaces, unmodified (a) and modified with APTES (b). Friction coefficient (defined as friction force/load) for studied systems (mean value: height of the colorful bars; standard deviation: vertical bars; median: horizontal bars) (c).

The averaged friction force measurements obtained at different increasing normal forces are shown in Figures 7a,b. The measurements on the pristine mica surface showed that the friction forces for polymers with epoxide and without an anchoring block were on the same level, while the friction forces for polymers with carboxyl and aldehyde groups were more than 50% lower over the entire range of the applied loads. Since the friction force increased linearly with the applied load, the friction coefficient, μ, was defined as the ratio between the friction force and the applied load. Figure 7c shows that the APTES modification had a significant impact on the lubrication properties. For all tested bottlebrush polymers, μ was lower on the APTES-modified mica surface compared to the pristine surface. On APTES-modified surfaces, the friction coefficient of the bottlebrush polymer without an anchoring block is significantly higher than for polymers able to anchor on the surface. The lowest friction coefficient on modified mica was registered for bottlebrushes with an epoxide anchoring group. This value is more than 95% lower than the friction coefficient for the same polymer deposited on the pristine mica surface. For polymers with other anchoring functions, the reduction of the friction coefficient in APTES-modified mica in relation to the pristine mica surface was around 80% for aldehyde and 76% for carboxylic groups, while for the polymer without an anchoring block, the friction coefficient was reduced only by 40%. These results indicate that the epoxide anchoring function provided the strongest and most stable binding of the bottlebrush polymer to the amine-functionalized surfaces. This observation is certainly linked to the formation of β-hydroxyamine resulting from the reaction of the epoxide groups with APTES primary amine function.^52^ As a consequence, this strong attachment could create a more stable lubricating layer, leading to the lowest friction coefficient. The aldehyde-anchored polymer exhibited a higher friction coefficient compared to the epoxide. This difference can be attributed to the weaker interactions formed between aldehydes and amines. Aromatic aldehydes are generally less reactive toward amines compared to epoxides due to the delocalized positive charge on the carbonyl carbon. As a result, efficient imine bond formation often requires acidic conditions, leading to weaker interactions and a less effective lubricating layer. However, they can still contribute to lubrication through hydrogen bonding or ionic interactions with surface amines and hydroxyl groups,^53^ resulting in a higher friction coefficient compared to epoxides but lower than nonanchoring polymers.

**Scheme 2:**
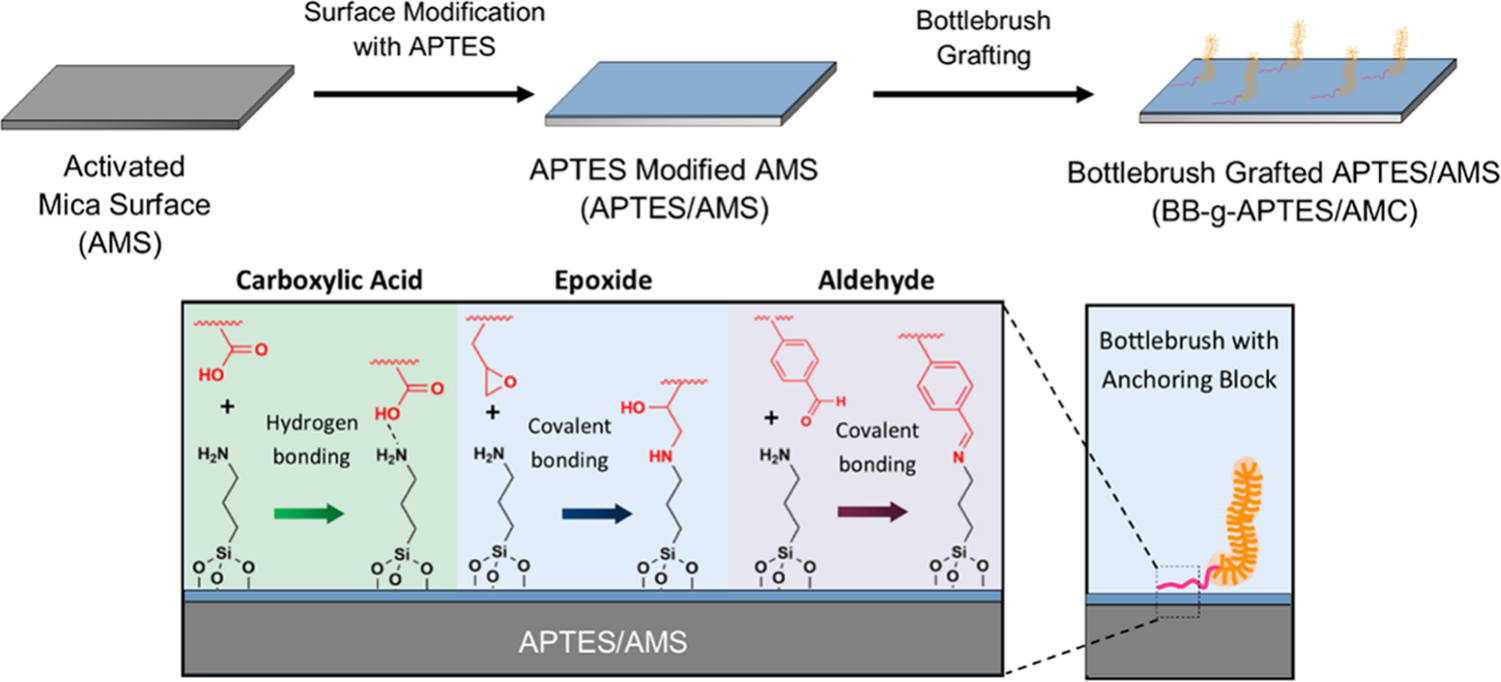
Anchoring of the Bottlebrush Polymers to the APTES-Modified Silicon Surface

Scheme 2 presents the possible covalent and physical bonds that could be formed during the adsorption of anchoring blocks with APTES-modified mica. In summary, different functional groups (carboxylic, aldehyde, and epoxide) within the bottlebrush polymer structure lead to varying lubrication effectiveness, likely due to their interaction with the amine-modified mica surface (mimicking cartilage). Finally, it is worthy to notice that, as we have shown recently,^54^ the lubrication properties of PMPC-containing bottlebrush polymers are less sensitive to ionic strength than polyelectrolyte brushes.^55^

**Figure 8:**
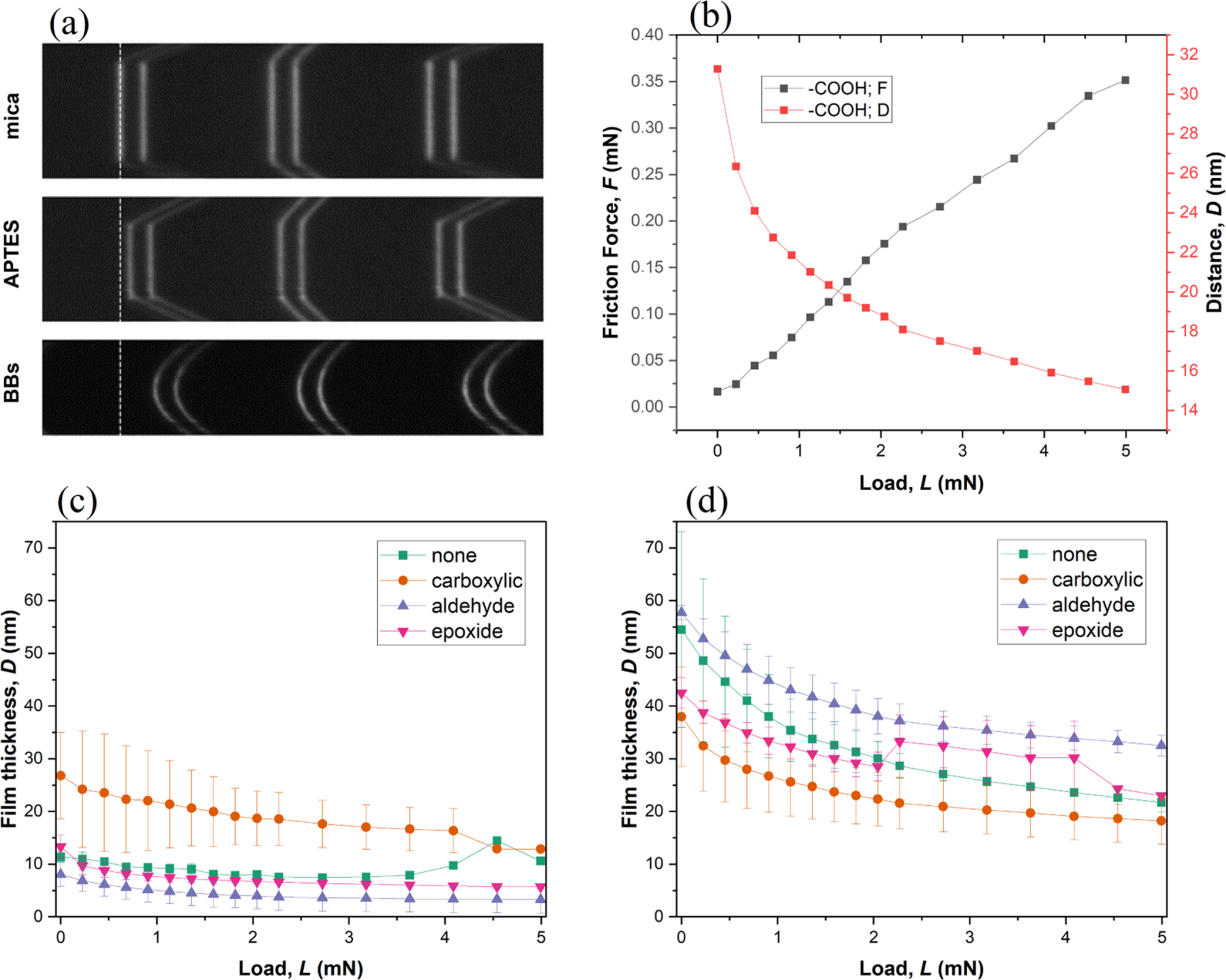
Fringes of equal chromatic order (FECO) in neat mica in air, mica modified with APTES, and mica with the polymer (BBs) deposited on the APTES layer (a), exemplary results of the thickness of the evolution of the polymer film and friction force under applied load for the polymer with a carboxylic anchoring group (b), film thickness evolution for all polymers on the mica surface (c), and film thickness evolution for all polymers on the APTES layer (d).

Examples of FECO images are shown in Figure 8a. When the surfaces were pressed against each other in air, flattening of the contact region was clearly observed for both the pristine mica and the APTES-modified mica. The shape of the FECO fringes observed in air for both types of surfaces is characteristic of a strongly adhesive contact.^56^ The comparison of FECO before and after surface modification with APTES enabled the quantification of the APTES layer thickness in air for each couple of shearing surfaces (≈10 nm). In the presence of bottlebrush polymers, the FECO fringes lost their flat shape even at the highest applied load (*L* = 5 mN, *P* = 10 MPa), which was attributed to the formation of a soft polymeric film. Throughout the lubrication tests, the film thickness of the polymer layer was monitored using the FECO to assess the load-bearing capacity of the different polymers. Figure 8b illustrates the representative examples of changes in both film thickness and friction force as a function of the applied load. The measured film thickness *D* on APTES-modified surfaces includes the thickness of the APTES layer as well (at loads over 1 mN, the APTES thickness in water was 10.2 ± 0.3 nm).

Under applied normal force, the compression of the polymer film resulted in a reduction of film thickness *D*, which correlated with an increase in surface friction force *F*. Figure 8c,d depicts the evolution of the film thickness under shear for pristine and modified mica, respectively, during the tribological experiment. The comparative analysis revealed that the APTES-modified surface exhibited significantly greater film thickness compared to neat mica, contributing to a reduction in the friction coefficient. This disparity in friction coefficients between the two mica types can be attributed to the improved interaction between the APTES-modified mica surface and the polymer molecules, as illustrated in Scheme 2. The increased efficacy of lubrication was observed for all polymer lubricants, with the epoxide functional groups showcasing optimal improvement on modified mica, resulting in a remarkable ninefold decrease in the friction coefficient from 0.27 to 0.03.

The characteristic power-law decay of *D* vs *L* for all the tested conditions (polymers and surfaces, Figure 8) is at the origin of the systematic linear relationship observed between *F* and *L* (Figure 7). Indeed, assuming that *L* is inversely proportional to *D* (*L* = *K*/*D*) and *F* is proportional to the Couette flow viscosity (*F* = η*V*/*D*) with *V* being the sliding speed and η being the thin film viscosity, the ratio *F*/*L* is proportional to *F*/*L* = η*V*/*K*, which is constant at constant shearing speed. This behavior was already reported for a triblock bottlebrush polymer with quaternized amine groups interacting with pristine mica surfaces via electrostatic interactions.^34^

#### 3.2.2 Anchoring on Articular Cartilage Tissue: Preliminary Ex Vivo Study

**Figure 9:**
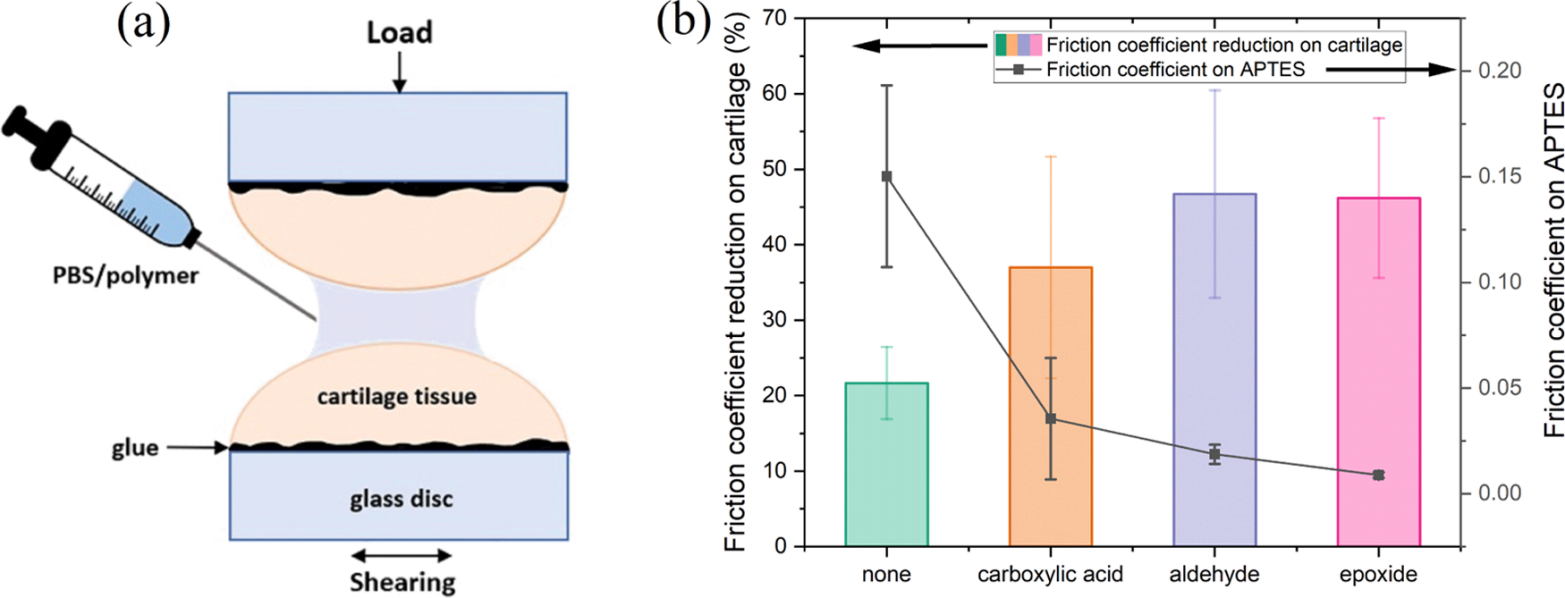
Lubrication test of bottlebrush polymers on cartilage tissue scheme (a) and comparison of friction coefficient reduction on chicken cartilage tissue and friction coefficient on APTES-modified mica (b).

To mimic the physiological conditions, the synthesized bottlebrush polymers were further tested on chicken cartilage. The articular cartilage consists of chondrocytes, highly specialized cells, and a dense extracellular matrix (ECM). The bulk of the ECM is composed of water, collagen, and proteoglycans, along with smaller amounts of noncollagenous proteins and glycoproteins. Since the frictional properties can vary greatly from one biological sample to another, the lubrication experiments were performed for each opposing couple of cartilage tissues first in PBS as a control condition and then with the polymer solution in the same contact (Figure 9a). The lubrication effectiveness of the bottlebrush polymers was quantified as the reduction in the friction coefficient compared to PBS (Figure 9b). For the polymer without the anchoring group, the friction coefficient decreased by 22%. In contrast, polymers with anchoring groups displayed higher lubrication capacity, with a reduction of friction coefficient ranging from 35 to 45% for carboxylic, aldehyde, and epoxide groups. Due to the high standard deviation, the differentiation of friction coefficient reduction among different functional groups in anchoring blocks necessitates cautious interpretation. The results demonstrated that all bottlebrush polymers, irrespective of their chemical structure, significantly reduced the friction coefficient between cartilage tissues, positioning them as promising biolubricants. Notably, bottlebrush polymers with anchoring blocks exhibited a greater reduction in the friction coefficient compared to those without anchoring blocks. This difference may be attributed to the nature of the interactions between the polymer and the surface zone of the cartilage, encompassing both physical and/or chemical interactions.

Comparative analysis of the results obtained on mica and cartilage (Figure 9b) revealed a robust correlation between both types of surfaces. The lower friction coefficient provided by the polymer on APTES-modified mica correlates with the higher efficiency of lubrication on the cartilage surface. This correlation underscores the potential translational relevance of findings from model surfaces to physiologically relevant substrates, strengthening the candidacy of these bottlebrush polymers for applications in joint lubrication.

## 4 Conclusions

The aim of this study was to investigate how the chemical functional groups in the anchoring blocks of bottlebrush polymers, resembling the structure of lubricin, influence their lubricating abilities. Three novel bottlebrush polymers with anchoring blocks containing carboxylic, epoxide, or aldehyde groups were synthesized using reversible-deactivation radical polymerization methods. Characterization of the polymers using SEC, NMR, DLS, and AFM techniques confirmed the successful synthetic procedures, resulting in well-defined bottlebrush topologies.

The lubricating properties and anchoring effectiveness were evaluated using the SFA on model surfaces (neat mica and APTES-modified mica). The friction forces measured on APTES-modified mica surfaces lubricated with bottlebrush polymers were significantly lower than those recorded on nonmodified mica surfaces. Measurements were conducted with a monoblock polymer lacking an anchoring block deposited as a reference on both bare mica and modified mica surfaces. While APTES-modified mica surfaces exhibited a 40% reduction in the friction coefficient for the reference polymer, diblock polymers with anchoring blocks demonstrated a 70–90% reduction in the friction coefficient on modified surfaces compared to the nonmodified mica. These results indicate that the anchoring groups can effectively interact with amine-functionalized surfaces via covalent or physical interactions. The lowest friction coefficient of 0.009 ± 0.001, with a 95% reduction compared to measurements on nonmodified mica, was observed for the polymer with an epoxide group in the anchoring block.

Preliminary lubrication tests performed on animal cartilage suggested that the anchoring blocks within the bottlebrush structure may influence its lubricating properties. While the reference polymer without anchoring groups only slightly reduced the friction coefficient, polymers capable of covalently binding to the rubbing surfaces demonstrated more significant reductions. Due to the diversity of biological materials, pinpointing the best-performing anchoring groups proved challenging. Nonetheless, preliminary experiments on chicken cartilage tissue indicated the potential of polymer bottlebrushes with anchoring blocks as cartilage lubricants for further in vivo studies.

## Supporting Information Available

^1^H NMR spectra of the synthesized monomers and polymer; FTIR spectra of polymers; and SEC graphs of polymers (PDF)

## ABBREVIATIONS

OA: osteoarthritis
HA: hyaluronic acid
RDRP: reversible-deactivation radical polymerization
RAFT: reversible addition–fragmentation chain transfer polymerization
ATRP: atom transfer radical polymerization
SFA: surface force apparatus
APTES: (3-aminopropyl)triethoxysilane
FPMA: 4-formylphenyl methacrylate
*t*BMA: *tert*-butyl methacrylate
GMA: glycidyl methacrylate
PMPC: poly(2-methacryloyloxyethyl phosphorylcholine)
FTIR: Fourier transform infrared spectroscopy
FECO: fringes of equal chromatic order

## References

[ref1] Huang X.; Ni B.; Mao Z.; Xi Y.; Chu X.; Zhang R.; Ma X.; You H. NOV/CCN3 Induces Cartilage Protection by Inhibiting PI3K/AKT/MTOR Pathway. J. Cell. Mol. Med. 2019, 23 (11), 7525–7534. 10.1111/jcmm.14621. 31454155 PMC6815824

[ref2] Wang M.; Shen J.; Jin H.; Im H. J.; Sandy J.; Chen D. Recent Progress in Understanding Molecular Mechanisms of Cartilage Degeneration during Osteoarthritis. Ann. N.Y. Acad. Sci. 2011, 61–69. 10.1111/j.1749-6632.2011.06258.x. PMC3671949 22172041

[ref3] Delplace V.; Boutet M.-A.; Visage C. L.; Maugars Y.; Guicheux J.; Vinatier C. Osteoarthritis: From Upcoming Treatments to Treatments yet to Come. Jt., Bone, Spine 2021, 88 (5), 105206 10.1016/j.jbspin.2021.105206. 33962030

[ref4] Lu K.; Ma F.; Yi D.; Yu H.; Tong L.; Chen D. Molecular Signaling in Temporomandibular Joint Osteoarthritis. J. Orthop. Transl. 2022, 21–27. 10.1016/j.jot.2021.07.001. PMC9072795 35591935

[ref5] Kuusalo L.; Felson D. T.; Wang N.; Lewis C. E.; Torner J.; Nevitt M. C.; Neogi T. Metabolic Osteoarthritis – Relation of Diabetes and Cardiovascular Disease with Knee Osteoarthritis. Osteoarthritis Cartilage 2021, 29 (2), 230–234. 10.1016/j.joca.2020.09.010. 33253888 PMC8020447

[ref6] Daste C.; Kirren Q.; Akoum J.; Lefèvre-Colau M.-M.; Rannou F.; Nguyen C. Physical Activity for Osteoarthritis: Efficiency and Review of Recommandations. Jt., Bone, Spine 2021, 88 (6), 105207 10.1016/j.jbspin.2021.105207. 33962031

[ref7] Khalid M.; Tufail S.; Aslam Z.; Butt A. Osteoarthritis: From Complications to Cure. Int. J. Clin. Rheumatol. 2017, 12 (6), 160–167. 10.4172/1758-4272.1000152.

[ref8] Richard M. J.; Driban J. B.; McAlindon T. E. Pharmaceutical Treatment of Osteoarthritis. Osteoarthritis Cartilage 2023, 31 (4), 458–466. 10.1016/j.joca.2022.11.005. 36414224

[ref9] Aweid O.; Osmani H.; Melton J. Biomechanics of the Knee. Orthop. Trauma 2019, 33 (4), 224–230. 10.1016/j.mporth.2019.05.004.

[ref10] Olinski M.; Gronowicz A.; Ceccarelli M. Development and Characterisation of a Controllable Adjustable Knee Joint Mechanism. Mech. Mach. Theory 2021, 155, 104101 10.1016/j.mechmachtheory.2020.104101.

[ref11] Maldonado M.; Nam J. The Role of Changes in Extracellular Matrix of Cartilage in the Presence of Inflammation on the Pathology of Osteoarthritis. BioMed Res. Int. 2013, 2013, 284873 10.1155/2013/284873. 24069595 PMC3771246

[ref12] Lin W.; Klein J. Recent Progress in Cartilage Lubrication. Adv. Mater. 2021, 33 (18), 2005513 10.1002/adma.202005513. 33759245

[ref13] Marian M.; Shah R.; Gashi B.; Zhang S.; Bhavnani K.; Wartzack S.; Rosenkranz A. Exploring the Lubrication Mechanisms of Synovial Fluids for Joint Longevity—A Perspective. Colloids Surf., B 2021, 206, 111926 10.1016/j.colsurfb.2021.111926. 34153619

[ref14] Zheng L.; Zhang Z.; Sheng P.; Mobasheri A. The Role of Metabolism in Chondrocyte Dysfunction and the Progression of Osteoarthritis. Ageing Res. Rev. 2021, 66, 101249 10.1016/j.arr.2020.101249. 33383189

[ref15] Rim Y. A.; Ju J. H. The Role of Fibrosis in Osteoarthritis Progression. Life 2021, 11, 3 10.3390/life11010003. PMC7822172 33374529

[ref16] Jahn S.; Seror J.; Klein J. Lubrication of Articular Cartilage. Annu. Rev. Biomed Eng. 2016, 18, 235–258. 10.1146/annurev-bioeng-081514-123305. 27420572

[ref17] An H.; Liu Y.; Yi J.; Xie H.; Li C.; Wang X.; Chai W. Research Progress of Cartilage Lubrication and Biomimetic Cartilage Lubrication Materials. Front. Bioeng. Biotechnol. 2022, 10, 1012653 10.3389/fbioe.2022.1012653. 36267457 PMC9576862

[ref18] Manasa C. S.; Silva S. M.; Desroches P. E.; Dennaoui J.; Russo M. J.; Han M.; Quigley A. F.; Greene G. W.; Kapsa R. M. I.; Moulton S. E. Lubricin as a Tool for Controlling Adhesion in Vivo and Ex Vivo. Biointerphases 2021, 16 (2), 020802 10.1116/6.0000779. 33736436

[ref19] Aninwene G. E. II; Abadian P. N.; Ravi V.; Taylor E. N.; Hall D. M.; Mei A.; Jay G. D.; Goluch E. D.; Webster T. J. Lubricin: A Novel Means to Decrease Bacterial Adhesion and Proliferation. J. Biomed. Mater. Res. A 2015, 103 (2), 451–462. 10.1002/jbm.a.35195. 24737699 PMC4669951

[ref20] Yuan H.; Mears L. L. E.; Wang Y.; Su R.; Qi W.; He Z.; Valtiner M. Lubricants for Osteoarthritis Treatment: From Natural to Bioinspired and Alternative Strategies. Adv. Colloid Interface Sci. 2023, 311, 102814 10.1016/j.cis.2022.102814. 36446286

[ref21] Peck J.; Slovek A.; Miro P.; Vij N.; Traube B.; Lee C.; Berger A. A.; Kassem H.; Kaye A. D.; Sherman W. F.; Abd-Elsayed A. A Comprehensive Review of Viscosupplementation in Osteoarthritis of the Knee. Orthop. Rev. 2021, 13, 25549 10.52965/001C.25549. PMC8567800 34745480

[ref22] Bowman E. N.; Hallock J. D.; Throckmorton T. W.; Azar F. M. Hyaluronic Acid Injections for Osteoarthritis of the Knee: Predictors of Successful Treatment. Int. Orthop 2018, 42 (4), 733–740. 10.1007/s00264-017-3731-8. 29299652

[ref23] Chavda S.; Rabbani S. A.; Wadhwa T. Role and Effectiveness of Intra-Articular Injection of Hyaluronic Acid in the Treatment of Knee Osteoarthritis: A Systematic Review. Cureus 2022, 14 (4), e24503 10.7759/cureus.24503. 35651409 PMC9135165

[ref24] Lakin B. A.; Cooper B. G.; Zakaria L.; Grasso D. J.; Wathier M.; Bendele A. M.; Freedman J. D.; Snyder B. D.; Grinstaff M. W. A Synthetic Bottle-Brush Polyelectrolyte Reduces Friction and Wear of Intact and Previously Worn Cartilage. ACS Biomater. Sci. Eng. 2019, 5 (6), 3060–3067. 10.1021/acsbiomaterials.9b00085. 31608307 PMC6788642

[ref25] Samaroo K. J.; Tan M.; Putnam D.; Bonassar L. J. Binding and Lubrication of Biomimetic Boundary Lubricants on Articular Cartilage. J. Orthop. Res. 2017, 35 (3), 548–557. 10.1002/jor.23370. 27419808

[ref26] Sun Z.; Bonassar L. J.; Putnam D. Influence of Block Length on Articular Cartilage Lubrication with a Diblock Bottle-Brush Copolymer. ACS Appl. Mater. Interfaces 2020, 12 (1), 330–337. 10.1021/acsami.9b18933. 31855406

[ref27] Charmi G.; Rahimi M.; Socha K.; Pham D. A.; Séguy L.; Phan Q. T.; Moldovan F.; Kozanecki M.; Matyjaszewski K.; Banquy X.; Pietrasik J. Bottlebrush Polymer with Dual Functionality for Osteoarthritis Treatment: Curcumin Delivery and Lubrication Properties. Polym. Chem. 2023, 14 (33), 3827–3833. 10.1039/D3PY00781B.

[ref28] Pham D. A.; Wang C. S.; Séguy L.; Zhang H.; Benbabaali S.; Faivre J.; Sim S.; Xie G.; Olszewski M.; Rabanel J. M.; Moldovan F.; Matyjaszewski K.; Banquy X. Bioinspired Bottlebrush Polymers Effectively Alleviate Frictional Damage Both In Vitro and In Vivo. Adv. Mater. 2024, 36, e2401689 10.1002/adma.202401689. 38552182

[ref29] Chiefari J.; Chong Y. K.; Ercole F.; Krstina J.; Jeffery J.; T Le T. P.; A Mayadunne R. T.; Meijs G. F.; Moad C. L.; Moad G.; Rizzardo E.; Thang S. H. Living Free-Radical Polymerization by Reversible Addition-Fragmentation Chain Transfer: The RAFT Process. Macromolecules 1998, 31, 5559–5562. 10.1021/ma9804951.

[ref30] Matyjaszewski K.; Xia J. Atom Transfer Radical Polymerization. Chem. Rev. 2001, 101 (9), 2921–2990. 10.1021/cr940534g. 11749397

[ref31] Martinez M. R.; Sobieski J.; Lorandi F.; Fantin M.; Dadashi-Silab S.; Xie G.; Olszewski M.; Pan X.; Ribelli T. G.; Matyjaszewski K. Understanding the Relationship between Catalytic Activity and Termination in PhotoATRP: Synthesis of Linear and Bottlebrush Polyacrylates. Macromolecules 2020, 53 (1), 59–67. 10.1021/acs.macromol.9b02397.

[ref32] Xie G.; Martinez M. R.; Olszewski M.; Sheiko S. S.; Matyjaszewski K. Molecular Bottlebrushes as Novel Materials. Biomacromolecules 2019, 20 (1), 27–54. 10.1021/acs.biomac.8b01171. 30296828

[ref33] Faivre J.; Shrestha B. R.; Xie G.; Delair T.; David L.; Matyjaszewski K.; Banquy X. Unraveling the Correlations between Conformation, Lubrication, and Chemical Stability of Bottlebrush Polymers at Interfaces. Biomacromolecules 2017, 18 (12), 4002–4010. 10.1021/acs.biomac.7b01063. 28960970

[ref34] Banquy X.; Burdyńska J.; Lee D. W.; Matyjaszewski K.; Israelachvili J. Bioinspired Bottle-Brush Polymer Exhibits Low Friction and Amontons-like Behavior. J. Am. Chem. Soc. 2014, 136 (17), 6199–6202. 10.1021/ja501770y. 24716507

[ref35] Maw M.; Morgan B. J.; Dashtimoghadam E.; Tian Y.; Bersenev E. A.; Maryasevskaya A. V.; Ivanov D. A.; Matyjaszewski K.; Dobrynin A. V.; Sheiko S. S. Brush Architecture and Network Elasticity: Path to the Design of Mechanically Diverse Elastomers. Macromolecules 2022, 55 (7), 2940–2951. 10.1021/acs.macromol.2c00006.

[ref36] Adibnia V.; Mirbagheri M.; Faivre J.; Robert J.; Lee J.; Matyjaszewski K.; Lee D. W.; Banquy X. Bioinspired Polymers for Lubrication and Wear Resistance. Prog. Polym. Sci. 2020, 110, 101298 10.1016/j.progpolymsci.2020.101298.

[ref37] Faivre J.; Shrestha B. R.; Xie G.; Olszewski M.; Adibnia V.; Moldovan F.; Montembault A.; Sudre G.; Delair T.; David L.; Matyjaszewski K.; Banquy X. Intermolecular Interactions between Bottlebrush Polymers Boost the Protection of Surfaces against Frictional Wear. Chem. Mater. 2018, 30 (12), 4140–4149. 10.1021/acs.chemmater.8b01676.

[ref38] García-Acosta B.; García F.; García J. M.; Martínez-Méñez R.; Sancenón F.; San-José N.; Soto J. Chromogenic Signaling of Hydrogen Carbonate Anion with Pyrylium-Containing Polymers. Org. Lett. 2007, 9 (13), 2429–2432. 10.1021/ol0705191. 17518473

[ref39] Negrell C.; Voirin C.; Boutevin B.; Ladmiral V.; Caillol S. From Monomer Synthesis to Polymers with Pendant Aldehyde Groups. Eur. Polym. J. 2018, 109, 544–563. 10.1016/j.eurpolymj.2018.10.039.

[ref40] Faivre J.; Shrestha B. R.; Burdynska J.; Xie G.; Moldovan F.; Delair T.; Benayoun S.; David L.; Matyjaszewski K.; Banquy X. Wear Protection without Surface Modification Using a Synergistic Mixture of Molecular Brushes and Linear Polymers. ACS Nano 2017, 11 (2), 1762–1769. 10.1021/acsnano.6b07678. 28071897

[ref41] Israelachvili J.; Min Y.; Akbulut M.; Alig A.; Carver G.; Greene W.; Kristiansen K.; Meyer E.; Pesika N.; Rosenberg K.; Zeng H. Recent Advances in the Surface Forces Apparatus (SFA) Technique. Rep. Prog. Phys. 2010, 73 (3), 036601 10.1088/0034-4885/73/3/036601.

[ref42] Kang T.; Banquy X.; Heo J.; Lim C.; Lynd N. A.; Lundberg P.; Oh D. X.; Lee H. K.; Hong Y. K.; Hwang D. S.; Waite J. H.; Israelachvili J. N.; Hawker C. J. Mussel-Inspired Anchoring of Polymer Loops That Provide Superior Surface Lubrication and Antifouling Properties. ACS Nano 2016, 10 (1), 930–937. 10.1021/acsnano.5b06066. 26695175 PMC4932843

[ref43] Banquy X.; Charrault E.; Giasson S. Normal and Lateral Interactions between Thermosensitive Nanoparticle Monolayers in Water. J. Phys. Chem. B 2010, 114 (30), 9721–9728. 10.1021/jp910965p. 20614943

[ref44] Banquy X.; Zhu X. X.; Giasson S. Mechanical and Frictional Properties of Nanoparticle Monolayers Grafted on Functionalized Mica Substrates. J. Phys. Chem. B 2008, 112 (39), 12208–12216. 10.1021/jp803605d. 18774849

[ref45] Monroy-Barreto M.; Esturau-Escofet N.; Briseño-Terán M.; del Carmen Pérez-Vázquez M. Microstructural Characterization and Thermal Analysis of Block Copolymer of Methyl Methacrylate and N-Butyl Acrylate. Int. J. Polym. Anal. Charact. 2012, 17 (7), 515–523. 10.1080/1023666X.2012.704556.

[ref46] Bustamante S. E.; Rivas B. L. New Synthesis Method to Obtain a Methacrylic Monomer with a Pyrylium Group. J. Chil. Chem. Soc. 2017, 62 (2), 3558–3561. 10.4067/S0717-97072017000200025.

[ref47] Zhao J.; Wang S.; Zhang L.; Wang C.; Zhang B. Kinetic, Isotherm, and Thermodynamic Studies for Ag(I) Adsorption Using Carboxymethyl Functionalized Poly(Glycidyl Methacrylate). Polymers 2018, 10 (10), 1090 10.3390/polym10101090. 30961015 PMC6403576

[ref48] Bayramoğlu G.; Yakup Arica M. Immobilization of Laccase onto Poly(Glycidylmethacrylate) Brush Grafted Poly(Hydroxyethylmethacrylate) Films: Enzymatic Oxidation of Phenolic Compounds. Mater. Sci. Eng. C 2009, 29 (6), 1990–1997. 10.1016/j.msec.2009.03.011.

[ref49] Maeda T.; Hagiwara K.; Yoshida S.; Hasebe T.; Hotta A. Preparation and Characterization of 2-Methacryloyloxyethyl Phosphorylcholine Polymer Nanofibers Prepared via Electrospinning for Biomedical Materials J. Appl. Polym. Sci. 2014 131 (14), 10.1002/app.40606.

[ref50] Rizzarelli E.; Theophanides T. Chemistry and Properties of Biomolecular Systems; Springer: Dordrecht, 1991.

[ref51] Christenson H. K.; Thomson N. H. The Nature of the Air-Cleaved Mica Surface. Surf. Sci. Rep. 2016, 71, 367–390. 10.1016/j.surfrep.2016.03.001.

[ref52] Ramsdale-Capper R.; Foreman J. P. Internal Antiplasticisation in Highly Crosslinked Amine Cured Multifunctional Epoxy Resins. Polymer 2018, 146, 321–330. 10.1016/j.polymer.2018.05.048.

[ref53] Chen J.; Murphy A. R.; Esteve J.; Ogletree D. F.; Salmeron M.; Fréchet J. M. J. Preparation and Nanoscale Mechanical Properties of Self-Assembled Carboxylic Acid Functionalized Pentathiophene on Mica. Langmuir 2004, 20 (18), 7703–7710. 10.1021/la030395a. 15323522

[ref54] Adibnia V.; Olszewski M.; De Crescenzo G.; Matyjaszewski K.; Banquy X. Superlubricity of Zwitterionic Bottlebrush Polymers in the Presence of Multivalent Ions. J. Am. Chem. Soc. 2020, 142 (35), 14843–14847. 10.1021/jacs.0c07215. 32790294

[ref55] Yu J.; Jackson N. E.; Xu X.; Morgenstern Y.; Kaufman Y.; Ruths M.; De Pablo J. J.; Tirrell M. Multivalent Counterions Diminish the Lubricity of Polyelectrolyte Brushes. Science 2018, 360, 1434–1438. 10.1126/science.aar5877. 29954973

[ref56] Horn R. G.; Israelachvili J. N.; Pribac F. Measurement of the Deformation and Adhesion of Solids in Contact. J. Colloid Interface Sci. 1987, 115 (2), 480–492. 10.1016/0021-9797(87)90065-8.

