# Supplementary material for: Bottlebrush Polymers for Articular Joint Lubrication: Influence of Anchoring Group Chemistry on Lubrication Properties
Source: ACS Appl Mater Interfaces. 2024 Jul 9;16(29):38550–63. doi: 10.1021/acsami.4c07282 (PMC11284745; doi:10.1021/acsami.4c07282)
Supplement: Supplementary file 1 — am4c07282_si_001.pdf [file am4c07282_si_001.pdf]

Supporting Information

Bottlebrush Polymers for Articular Joints Lubrication: Influence of Anchoring Groups Chemistry on Lubrication Properties

Karolina Turczyńska[a]‡, Mahdi Rahimi[b]‡, Gholamreza Charmi[c]‡, Duy Anh Pham[d], Hironobu Murata[e], Marcin Kozanecki[a], Paulina Filipczak[a], Jacek Ulański[a], Tadeusz Diem[f], Krzysztof Matyjaszewski[a,e], Xavier Banquy[d]*, Joanna Pietrasik[c]*

[a] *Department of Molecular Physics, Faculty of Chemistry, Lodz University of Technology, Zeromskiego 116, 90-924 Lodz, Poland*

[b] *Orthopedic Research Laboratory, Hôpital du Sacré-Cœur de Montréal, Université de Montréal, Montréal, QC, Canada*

[c] *Institute of Polymer and Dye Technology, Faculty of Chemistry, Lodz University of Technology, Stefanowskiego 16, 90-537 Lodz, Poland*

[d] *Canada Research Chair in Bio-inspired Materials and Interfaces, Faculty of Pharmacy, Université de Montréal C.P. 6128, succursale Centre Ville, Montréal, H3T1J4 QC, Canada*

[e] *Department of Chemistry, Carnegie Mellon University, 4400 Fifth Avenue, Pittsburgh, Pennsylvania 15213, United States*

[f] *Collegium Civitas, Plac Defilad 1, 00-901 Warsaw, Poland*

Email: xavier.banquy@umontreal.ca; joanna.pietrasik@p.lodz.pl

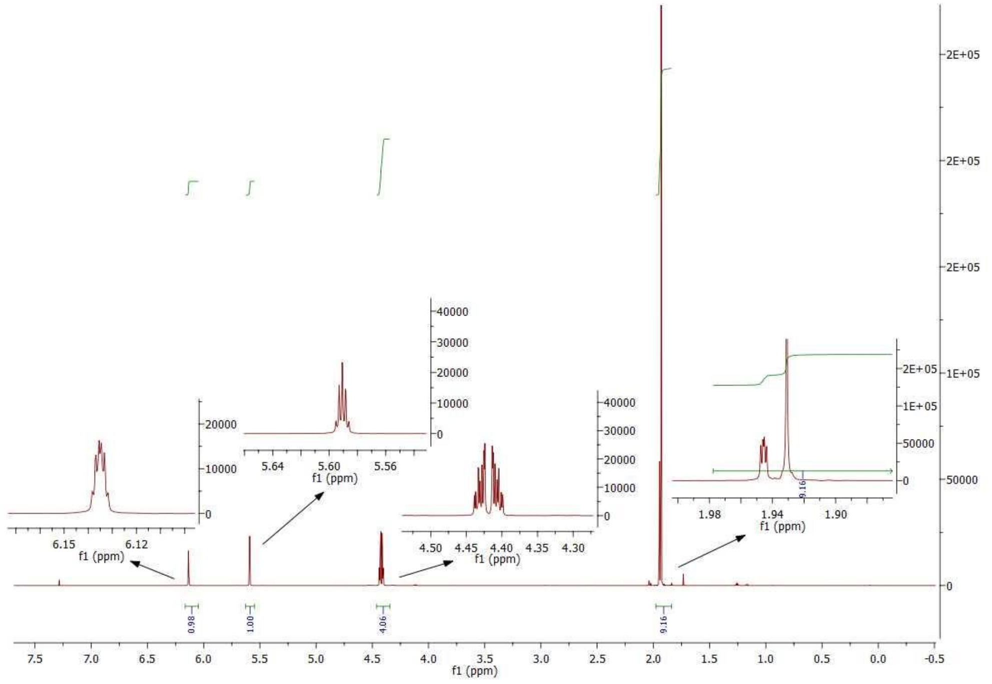

Figure S1. The ^{1}H NMR spectra of BIBEMA monomer in $CDCl_3$.

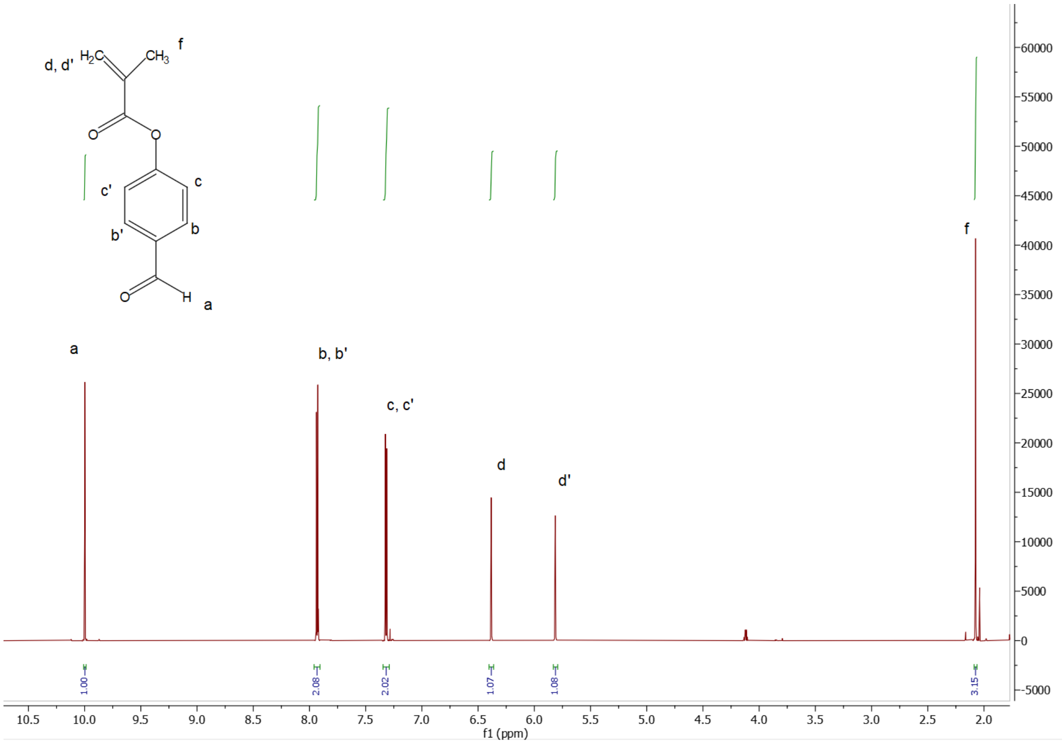

Figure S2. The ^{1}H NMR spectra of FPMA monomer in $CDCl_3$

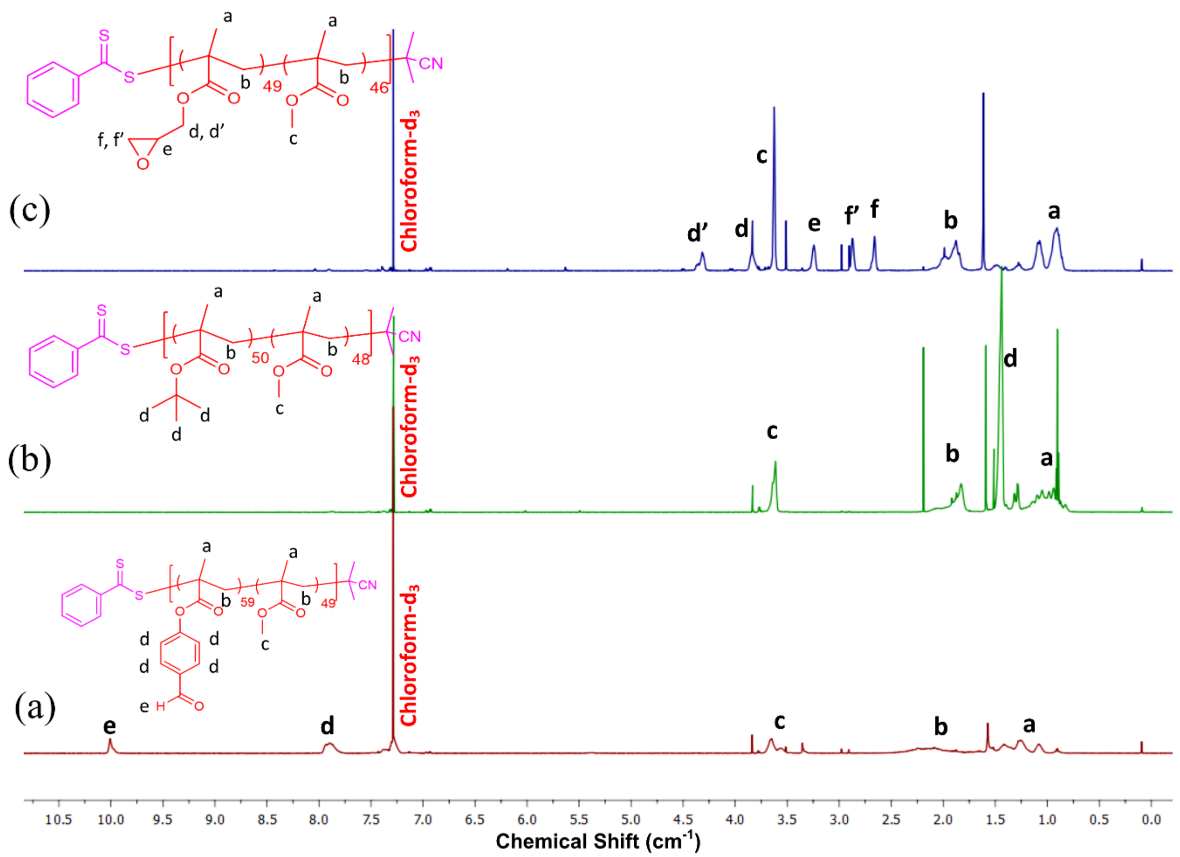

Figure S3. The ^{1}H NMR spectra of P(FBMA$_{59}$-*co*-MMA$_{49}$) (**a**), P(*t*BMA$_{50}$-*co*-MMA$_{48}$) (**b**), and P(GMA$_{49}$-*co*-MMA$_{46}$) (**c**) in chloroform- d$_{3}$.

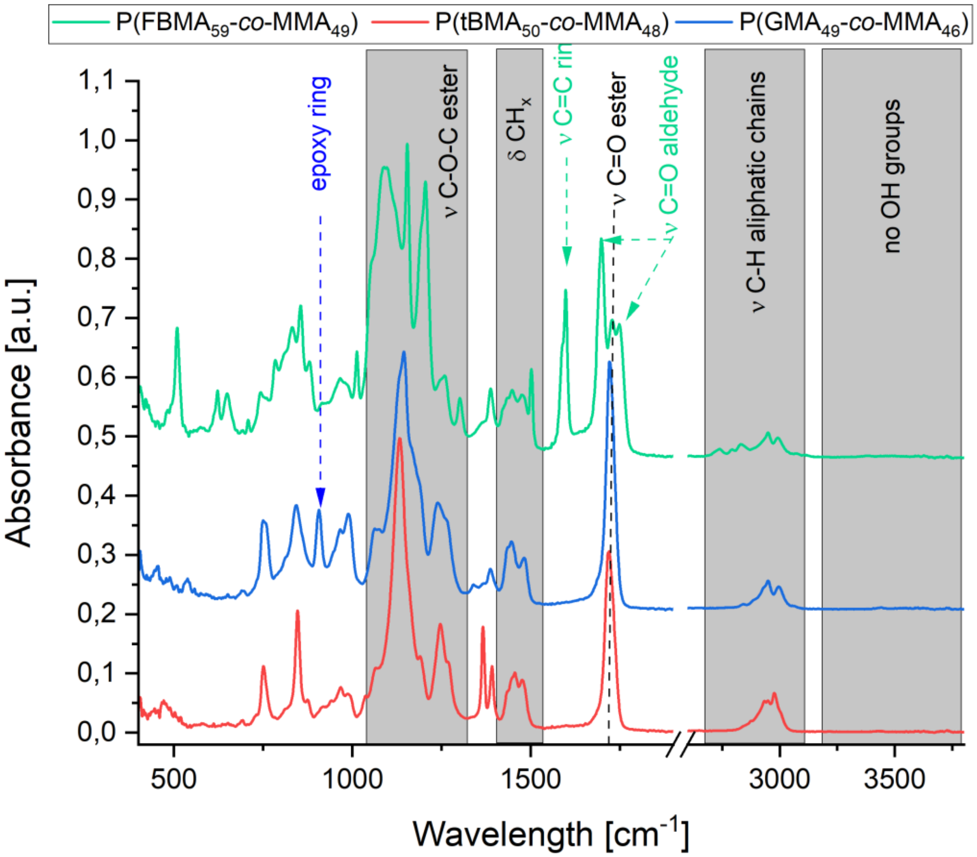

Figure S4. The FTIR spectra of P(FBMA$_{59}$-*co*-MMA$_{49}$), P(*t*BMA$_{50}$-*co*-MMA$_{48}$), and P(GMA$_{49}$-*co*-MMA$_{46}$) in the wavenumber ranges of 400–3800 cm^{-1}. The bands important for discussion were assigned according to literature values.[1-4]

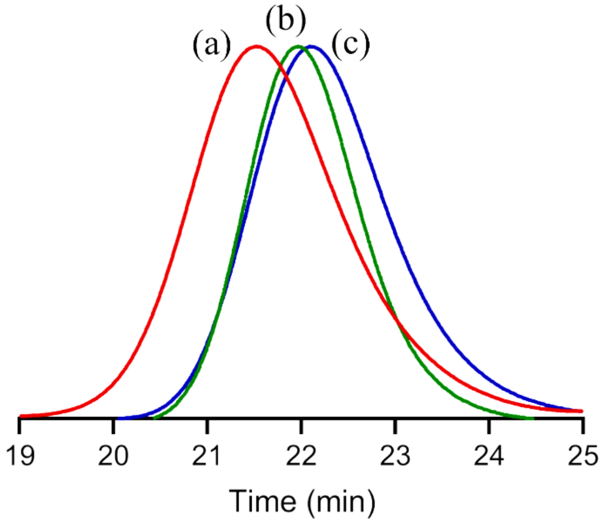

Figure S5. The SEC graph of P($FBMA_{59}$-*co*-MMA_{49}), M_n=13,200 (**a**), (P*t*BMA_{50}-*co*-MMA_{48}) M_n=11,600 (**b**), and P(GMA_{49}-*co*-MMA_{46}) M_n=9,000 (**c**) using linear PMMA standards in DMF.

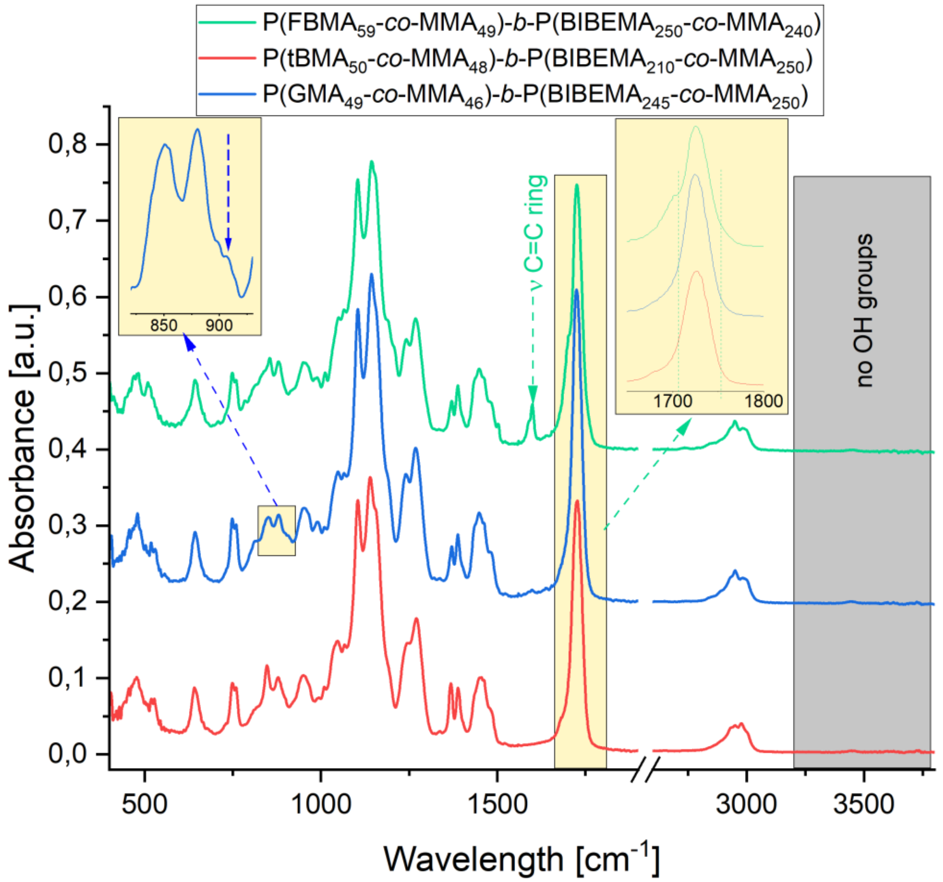

Figure S6. The FTIR spectra of P(FBMA$_{59}$-*co*-MMA$_{49}$)-*b*-P(BIBEMA$_{250}$-*co*-MMA$_{240}$), P(*t*BMA$_{50}$-*co*-MMA$_{48}$)-*b*-P(BIBEMA$_{210}$-*co*-MMA$_{250}$), and P(GMA$_{49}$-*co*-MMA$_{46}$)-*b*-P(BIBEMA$_{245}$-*co*-MMA$_{250}$) in the wavenumber ranges of 400–3800 cm^{-1}. The inset in the range 820-930 cm^{-1} shows the region of the line characteristic for epoxide group (907 cm^{-1}) marked by arrow. The inset in the range 1650-1800 cm^{-1} shows the region of C=O stretching – positions of lines characteristic for aldehydes are marked by vertical dashed lines.

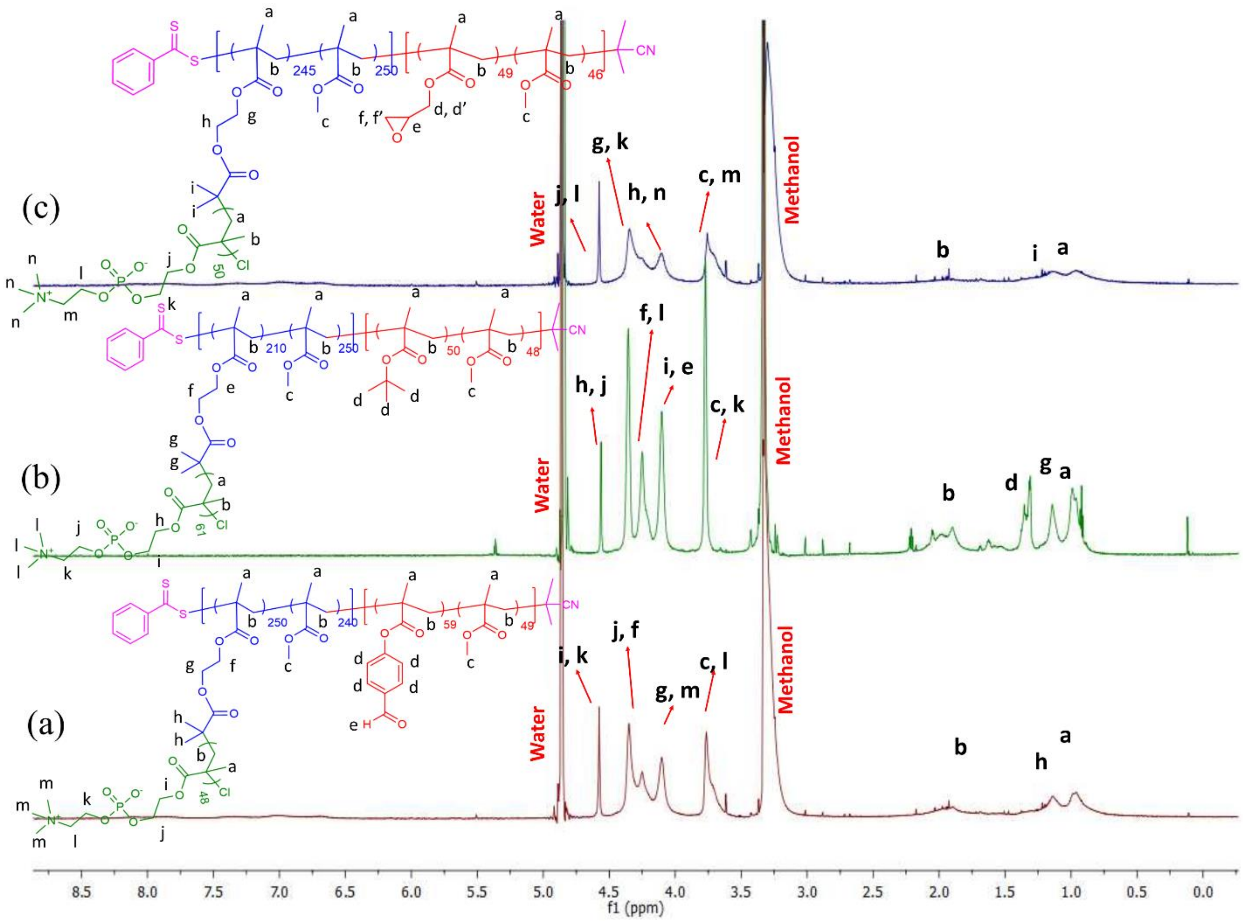

Figure S7. The ^{1}H NMR spectra of P(FBMA$_{59}$-*co*-MMA$_{49}$)-*b*-(P(BIBEMA$_{250}$-*co*-MMA$_{240}$)-*g*-PMPC$_{48}$) (**a**), P(*t*BMA$_{50}$-*co*-MMA$_{48}$)-*b*-(P(BIBEMA$_{210}$-*co*-MMA$_{250}$)-*g*-PMPC$_{61}$) (**b**), and P(GMA$_{49}$-*co*-MMA$_{46}$)-*b*-(P(BIBEMA$_{245}$-*co*-MMA$_{250}$)-*g*-PMPC$_{50}$) (**c**) in methanol-d$_4$.

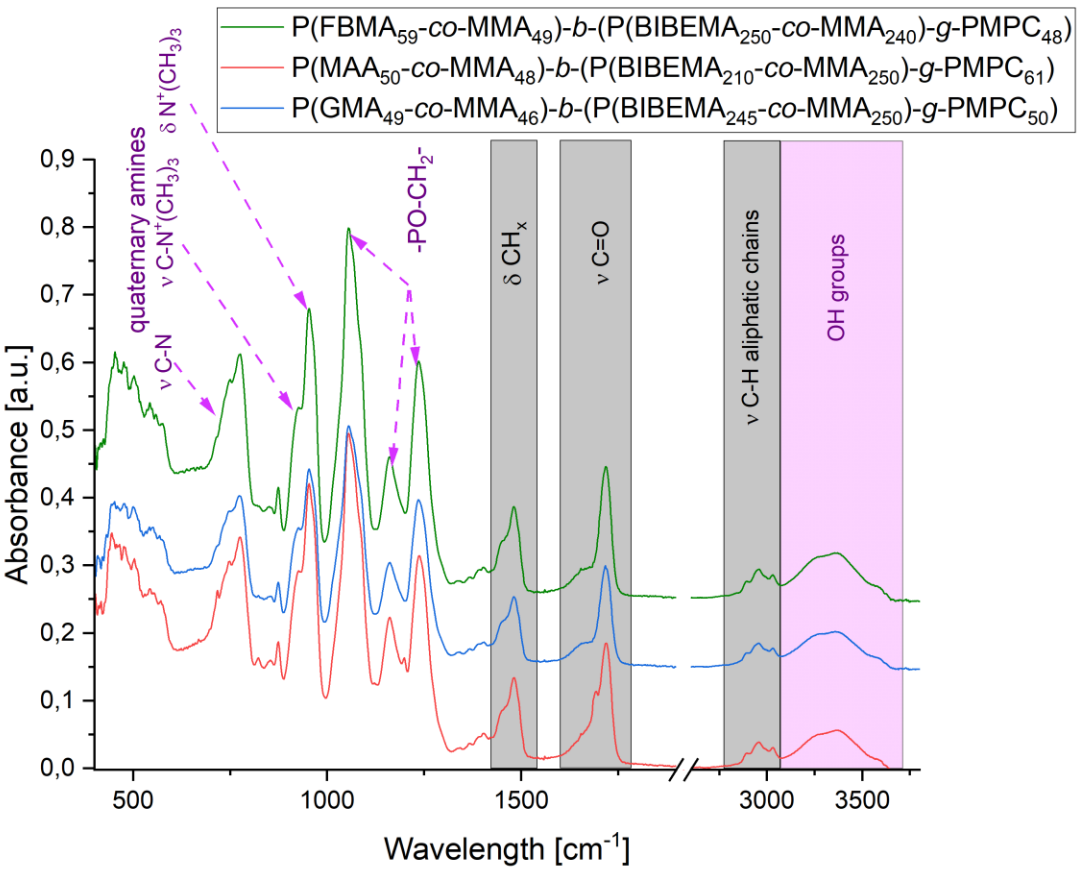

Figure S8. FTIR spectra of P(BIBEMA$_{246}$-*co*-MMA$_{249}$)-*g*-PMPC$_{52}$ P(FBMA$_{59}$-*co*-MMA$_{49}$)-*b*-(P(BIBEMA$_{250}$-*co*-MMA$_{240}$)-*g*-PMPC$_{48}$), P(MAA$_{50}$-*co*-MMA$_{48}$)-*b*-(P(BIBEMA$_{210}$-*co*-MMA$_{250}$)-*g*-PMPC$_{61}$) , and P(GMA$_{49}$-*co*-MMA$_{46}$)-*b*-(P(BIBEMA$_{245}$-*co*-MMA$_{250}$)-*g*-PMPC$_{50}$).

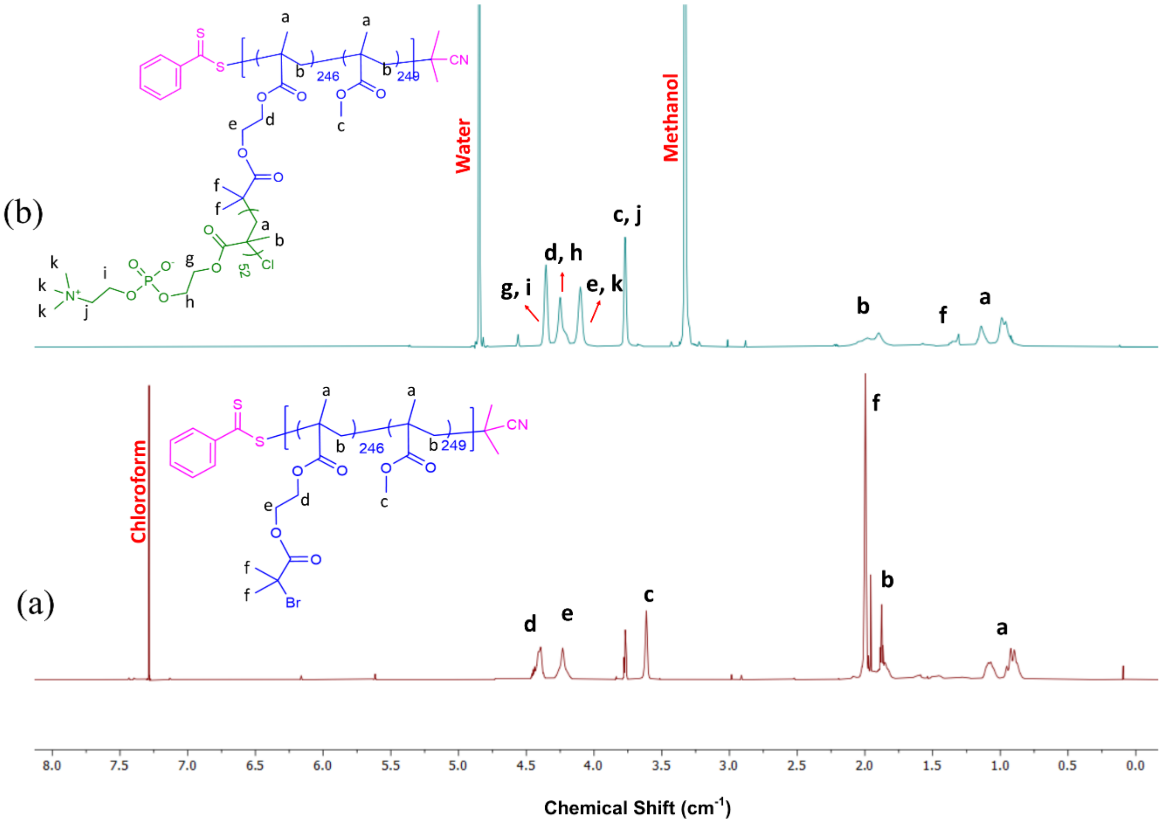

Figure S9: The ^{1}H NMR spectra of the P(BIBEMA$_{246}$-*co*-MMA$_{249}$) in chloroform- d$_3$ (**a**) and P(BIBEMA$_{246}$-*co*-MMA$_{249}$)-*g*-PMPC$_{52}$ in methanol- d$_4$ (**b**).

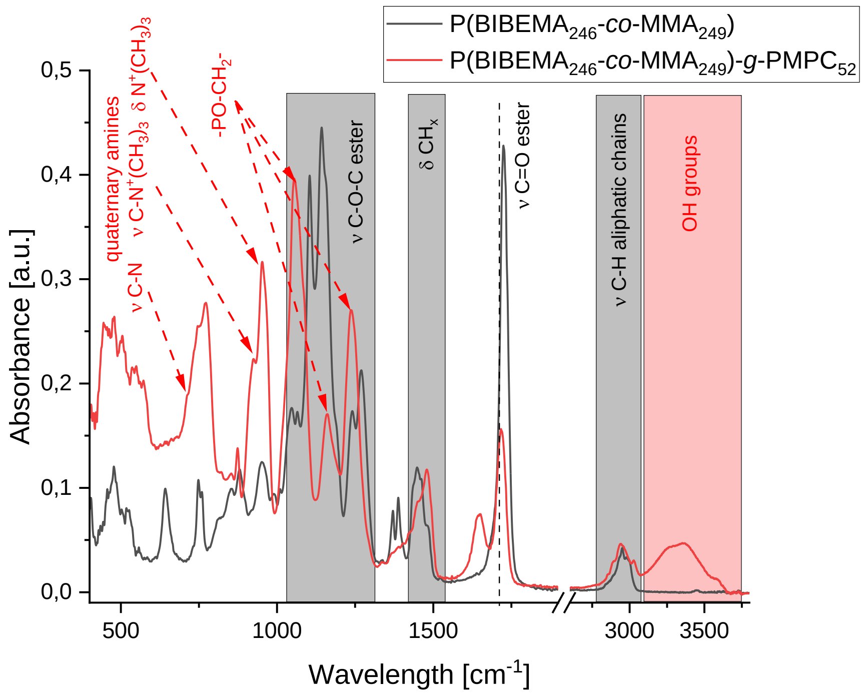

Figure S10. The FTIR spectra of P(BIBEMA$_{246}$-*co*-MMA$_{249}$) and P(BIBEMA$_{246}$ -*co*-MMA$_{249}$)-*g*-PMPC$_{52}$ in the wavenumber ranges of 500–4000 cm^{-1}.

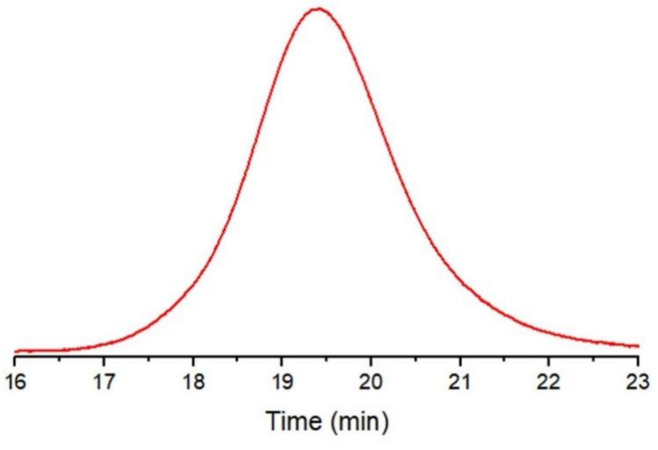

Figure S11. The SEC graph of P(BIBEMA$_{246}$-*co*-MMA$_{249}$) using linear PMMA standards in DMF; M_n=53,400 g/mol.

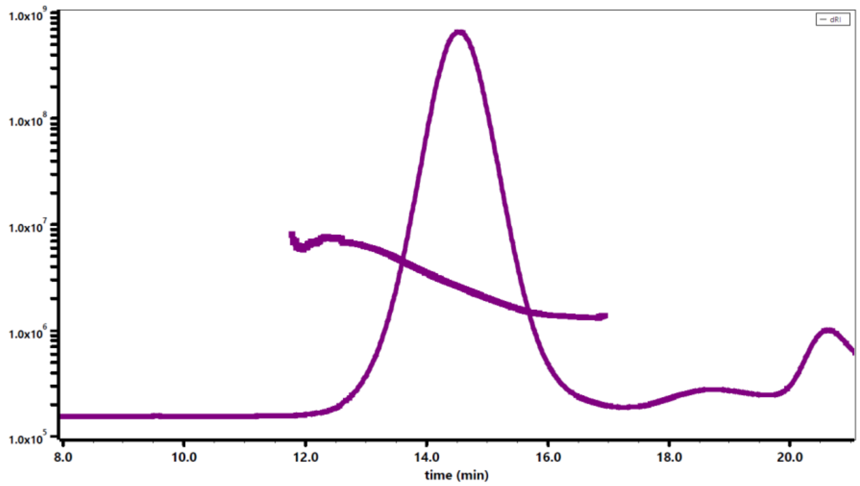

Figure S12. The SEC-MALS measurement of P(BIBEMA$_{246}$-*co*-MMA$_{249}$)-*g*-PMPC$_{52}$ using PBS buffer (pH 7.4) and 100 mM sodium phosphate (pH 2.5) with 0.2 vol% of trifluoroacetic acid as eluent; M_n=3,632,000 g/mol.

References:

(1) Monroy-Barreto, M.; Esturau-Escofet, N.; Briseño-Terán, M.; del Carmen Pérez-Vázquez, M. Microstructural Characterization and Thermal Analysis of Block Copolymer of Methyl Methacrylate and N-Butyl Acrylate. *International Journal of Polymer Analysis and Characterization* **2012**, *17* (7), 515–523, DOI: 10.1080/1023666X.2012.704556.

(2) Bustamante, S. E.; Rivas, B. L. New Synthesis Method to Obtain a Methacrylic Monomer with a Pyrylium Group. *J. Chil. Chem. Soc* **2017**, *62* (2), DOI: doi.org/10.4067/S0717-97072017000200025.

(3) Zhao, J.; Wang, S.; Zhang, L.; Wang, C.; Zhang, B. Kinetic, Isotherm, and Thermodynamic Studies for Ag(I) Adsorption Using Carboxymethyl Functionalized Poly(Glycidyl Methacrylate). *Polymers* **2018**, *10* (10), DOI: 10.3390/polym10101090.

(4) Bayramoğlu, G.; Yakup Arica, M. Immobilization of Laccase onto Poly(Glycidylmethacrylate) Brush Grafted Poly(Hydroxyethylmethacrylate) Films: Enzymatic Oxidation of Phenolic Compounds. *Materials Science and Engineering C* **2009**, *29* (6), 1990–1997, DOI: 10.1016/j.msec.2009.03.011.
